# Multimodality Cardiac Imaging for Procedural Planning and Guidance of Transcatheter Mitral Valve Replacement and Mitral Paravalvular Leak Closure

**DOI:** 10.3389/fcvm.2021.582925

**Published:** 2021-02-22

**Authors:** Enrique Garcia-Sayan, Tiffany Chen, Omar K. Khalique

**Affiliations:** ^1^Division of Cardiology, University of Texas Health Science Center at Houston, Houston, TX, United States; ^2^Division of Cardiovascular Medicine, Perelman School of Medicine, University of Pennsylvania, Philadelphia, PA, United States; ^3^Division of Cardiology, Structural Heart and Valve Center, Columbia University Medical Center, New York, NY, United States

**Keywords:** transcatheter mitral valve replacement, paravalvular leak closure, multimodality cardiac imaging, cardiovascular computed tomography angiography, 3-dimentional transesophageal echocardiography, valve-in-valve, valve-in-ring, valve-in-MAC

## Abstract

Transcatheter mitral valve interventions are an evolving and growing field in which multimodality cardiac imaging is essential for diagnosis, procedural planning, and intraprocedural guidance. Currently, transcatheter mitral valve-in-valve with a balloon-expandable valve is the only form of transcatheter mitral valve replacement (TMVR) approved by the FDA, but valve-in-ring and valve-in-mitral annular calcification interventions are increasingly being performed. Additionally, there are several devices under investigation for implantation in a native annulus. Paravalvular leak (PVL) is a known complication of surgical or transcatheter valve implantation, where regurgitant flow occurs between the prosthetic sewing ring and the native mitral annulus. We sought to describe the role and applications of multimodality cardiac imaging for TMVR, and PVL closure, including the use of Cardiovascular Computed Tomography Angiography and 3-Dimensional Transesophageal Echocardiography for diagnosis, prosthetic valve evaluation, pre-procedural planning, and intraprocedural guidance, as well as evolving technologies such as fusion imaging and 3D printing.

**Graphical Abstract:**
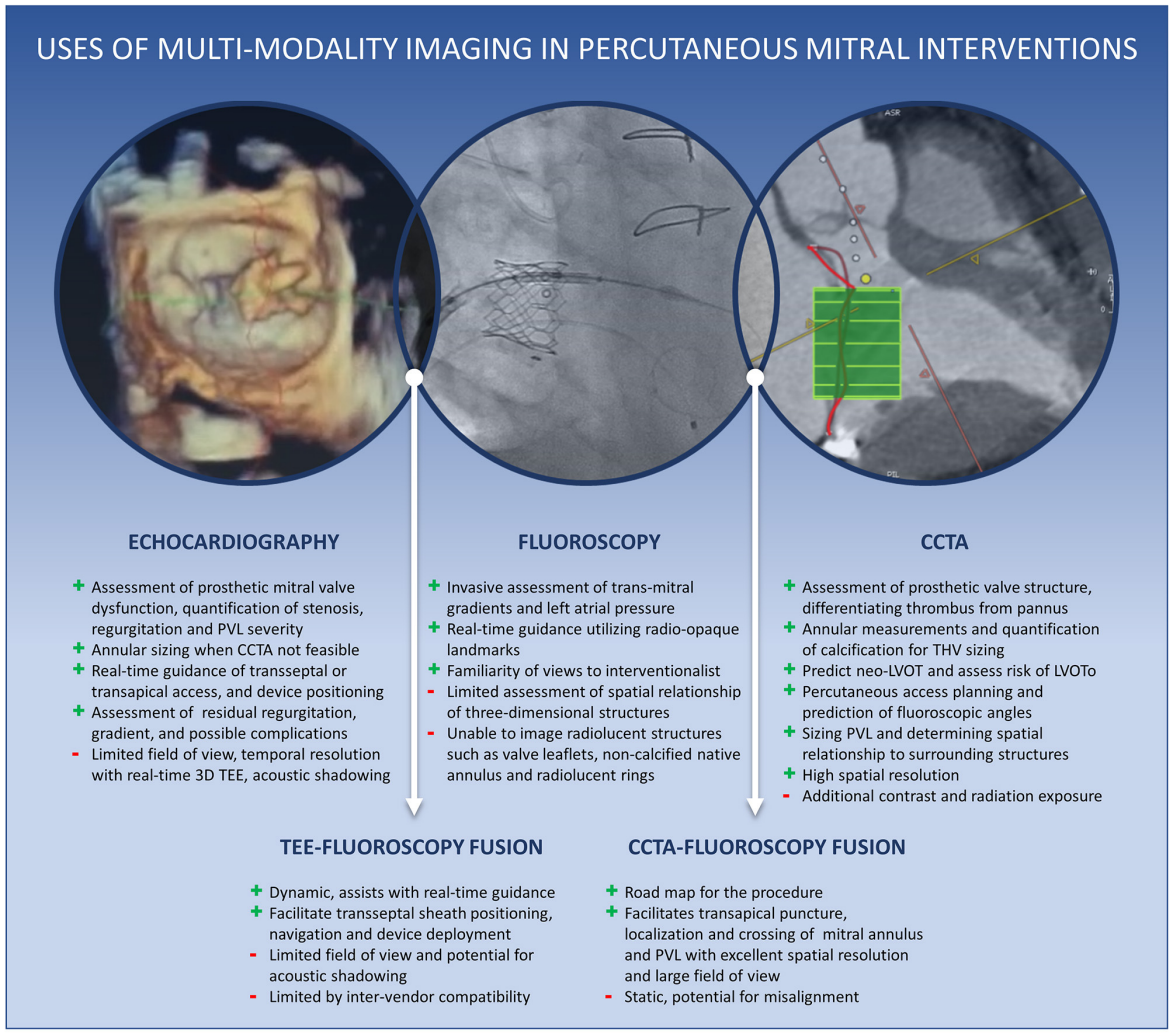
Central Illustration. Uses of multimodality cardiac imaging in percutaneous mitral interventions.

## Introduction

With the approval of transcatheter mitral valve-in-valve therapy by the United States Food and Drug Administration (FDA) in 2017 and several devices under clinical investigation for native mitral disease, transcatheter mitral interventions are the next rapidly evolving frontier after the unabated success of transcatheter aortic valve replacement. Structural valve dysfunction (SVD) is common in mitral bioprostheses and can lead to significant regurgitation (most common, 49%), stenosis (21%), or both (30%) as the mechanism of failure (1). Furthermore, mitral annuloplasty repair is associated with high rates of recurrent mitral regurgitation (MR), especially in those with functional MR. Many patients with degenerated mitral bioprostheses or rings who develop SVD are at high, if not prohibitive, risk for a redo operation due to older age and multiple co-morbidities (2). Also common in the older population with poor surgical outcomes is degenerative mitral valve disease due to severe mitral annular calcification (MAC). Therefore, valve-in-valve (ViV), valve-in-ring (ViR), and valve-in-mitral annular calcification (ViMAC) TMVR have emerged as increasingly valuable percutaneous alternatives to surgical therapy in this population. Paravalvular leak (PVL) is another form of prosthetic mitral dysfunction that is amenable to percutaneous therapy. Advanced cardiac imaging is an integral part of the evaluation and guidance of transcatheter transmitral procedures. This review will focus on multimodality cardiac imaging for TMVR and PVL closure in terms of preprocedural evaluation and intraprocedural guidance.

## Preprocedural Evaluation

The role of imaging in the pre-procedural planning of TMVR or PVL closure involves identifying the mechanism of valvular dysfunction, quantifying the severity of mitral regurgitation or stenosis, assessing mitral anatomy (native or prosthetic), and evaluating vascular access for transcatheter device delivery. Given the strengths and limitations of different imaging techniques in accomplishing each of these tasks, TMVR planning requires multimodality imaging, predominantly echocardiography and cardiovascular computed tomography angiography (CCTA). Hence, an advanced cardiovascular imager specializing in these particular modalities is considered an integral member of the structural heart team (3).

### Assessment of Prosthetic Mitral Valve Function

**Figure 1:**
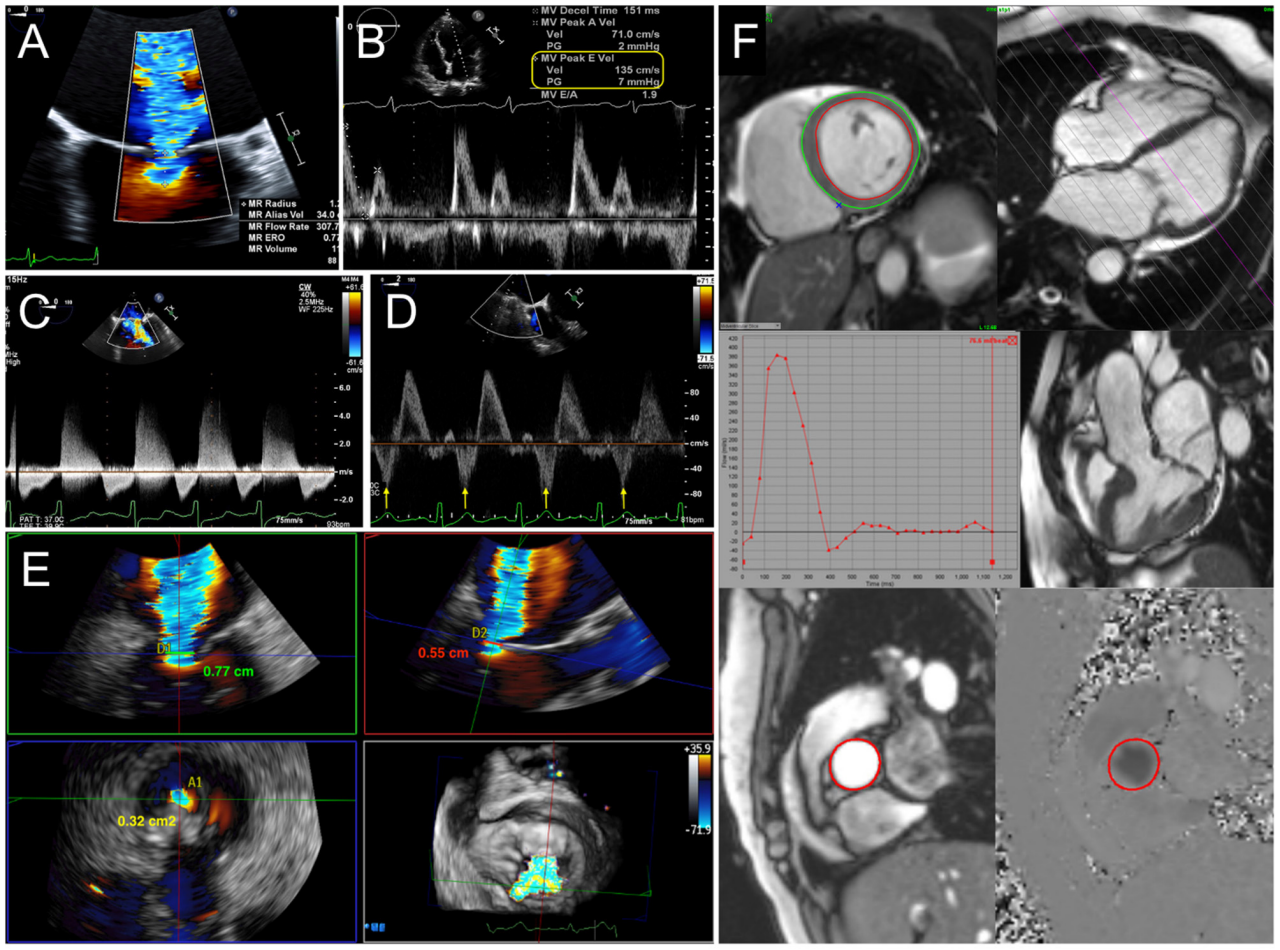
Quantification of mitral regurgitation (MR). **(A)** Calculation of effective regurgitant orifice area (EROA) and regurgitant volume (RVol) by proximal isovelocity surface area (PISA) method. Indicators for severe MR include elevated early diastolic mitral inflow (E wave) velocity **(B)**, dense continuous-wave Doppler signal **(C)**, and systolic reversal of pulmonary vein flow **(D)**. **(E)** 3D vena contracta area reconciles discrepant linear vena contracta width measurements in an elliptical regurgitant orifice. **(F)** With cardiac MRI, mitral RVol is calculated as the difference between total left ventricular stroke volume and the aortic flow (forward stroke volume) by phase-contrast imaging.

Assessment of prosthetic mitral valve function relies primarily on echocardiography, particularly transesophageal echocardiography (TEE), for identifying the mechanism of failure and quantifying the degree of regurgitation or stenosis. As with the quantification of native regurgitation, the severity of prosthetic MR is assessed by an integrative, multiparametric approach (4, 5). Standard methods for qualitative and quantitative assessment of MR incorporate continuous wave Doppler, jet area by color Doppler, vena contracta width (VCW), and the approximation of the effective regurgitant orifice area (EROA) and regurgitant volume by the proximal isovelocity surface area (PISA) method. However, non-circular and eccentric regurgitant jets can be better assessed by either three-dimensional echocardiography (3DE), which allows for direct planimetry of the vena contracta area (VCA) (6, 7), or volumetric methods by Doppler echocardiography or cardiac magnetic resonance (CMR). Finally, secondary signs of significant MR, such as left atrial (LA) and left ventricular (LV) dilatation, pulmonary hypertension, and systolic reversal of pulmonary vein flow, should also be assessed (Figure 1).

Evaluation for prosthetic mitral stenosis (MS) by echocardiography involves transvalvular peak velocity, gradients, and pressure half-time (PHT), (8), which can all be influenced by heart rate, loading conditions, and differences in the relative compliances of LA and LV that may be less reliable in the elderly population. In contrast, the effective orifice area (EOA) calculated by continuity equation and velocity-time integral (VTI) ratio between mitral inflow and LV outflow tract (LVOT) forward stroke volume are relatively flow-independent parameters, but are less reliable in the presence of significant valvular regurgitation. Reduction in EOA is suggestive of valve obstruction but may result from patient-prosthesis mismatch (PPM), which is not as commonly encountered in mitral prostheses as aortic valves. Differentiation between prosthetic stenosis and PPM relies upon the presence of abnormal leaflet thickening and motion in the former, in addition to the time course of changes in valve hemodynamics and clinical context. Although TEE offers higher temporal resolution (9) and 3D TEE can allow for accurate measurements of mitral annular and valve dimensions, image quality can be limited by acoustic shadowing. Multiphasic (4D) CCTA can be very helpful in further evaluating prosthetic valve structure, positioning, leaflet motion, and extent of calcification.

### Etiology of Prosthetic Valve Failure

Suitability for ViV TMVR involves not only the detection of significant MR or MS but also the elucidation of the mechanism of prosthetic valve failure and confirmation that the pathologic regurgitation is intravalvular rather than paravalvular. Although SVD is the most common mode of bioprosthetic mitral valve failure, endocarditis and thrombosis should also be considered and thoroughly evaluated with TEE and other imaging modalities. Exclusion of infective endocarditis is paramount since TMVR would be contraindicated. Positron emitted tomography (PET) can be used as an adjunctive tool for detecting paravalvular abscess in cases of ambiguity (10). Valve thrombosis is more prevalent in mechanical, rather than biologic, mitral prostheses, although the prevalence in bioprosthetic valves may be under-recognized (11, 12). Nonetheless, thrombus formation of bioprosthetic mitral valves can occur and can be detected by TEE as a soft echodensity with impairment of leaflet motion and increased gradients. In contrast, pannus is characterized by a more echogenic appearance, limited mobility, and predilection for the sewing ring. CCTA is an excellent modality to not only evaluate prosthetic valve leaflet motion but also differentiate thrombus from pannus, based on differences in attenuation (9). Valve thrombosis is important to identify since there may be a role for fibrinolytic or escalated anticoagulant therapy prior to consideration for ViV TMVR.

### Paravalvular Regurgitation and Implications for PVL Closure

PVL is defined as flow between the prosthetic sewing ring and the native mitral annulus. The presence of PVL is nearly always pathologic and results from (a) inadequate suture anchoring due to local calcification or friable tissue or (b) partial dehiscence due to endocarditis, inflammation, or degenerative causes. Clinically, PVL presents similarly to other causes of prosthetic valve dysfunction with heart failure symptoms but can also result in significant hemolytic anemia. In mitral annuloplasty rings, PVL usually refers to ring dehiscence and worsening of intravalvular regurgitation. Extravalvular, para-ring regurgitation implies perforation in the native leaflets.

**Figure 2:**
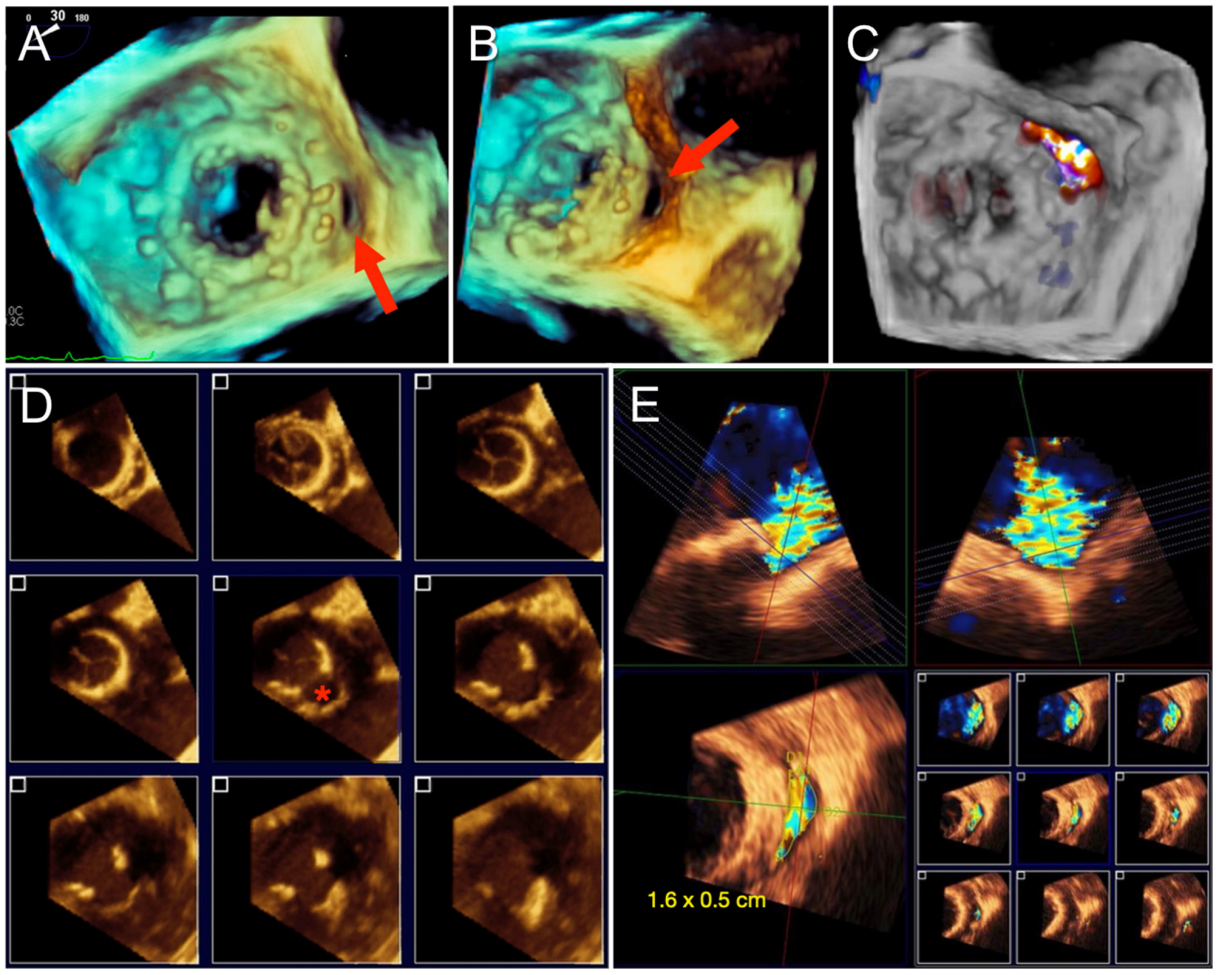
Paravalvular mitral regurgitation assessment by TEE. **(A)** 3D *en face* “surgeon's view” of a mitral bioprosthesis demonstrates a gap along the posteromedial aspect, corresponding to a paravalvular defect. **(B)** Rotation of the 3D volume improves visualization of the paravalvular defect. **(C)** Color Doppler confirms regurgitant flow through the defect and no other areas of paravalvular leak (PVL). Sizing of PVL by TEE can be difficult due to irregularity in the shape of the defect and dropout artifact, as shown in **(D)**, and is best measured with color Doppler to delineate the regurgitant PVL jet and avoid overestimation **(E)**.

Quantification of PVL severity can be more difficult than intravalvular MR due to acoustic shadowing of the paravalvular tract (especially from mechanical valves) and the tendency for jets to be thin and crescent-shaped with an eccentric trajectory. Therefore, it is especially important to interrogate the valve systematically from multiple views, integrate other findings including pulmonary vein flow, and to utilize 3D VCA to quantify eccentric and non-circular jets. The proportion of the circumference of the sewing ring occupied by the PVL is utilized as an indication of severity (5). A greater extent may be associated with instability of the prosthesis (“rocking”) and worse outcomes with percutaneous PVL closure (13). Planning for transcatheter PVL closure involves considering not only the size of the defect but also its location. Conventional mitral PVL localization is based on a clock-face configuration of the *en face* “surgeon's view” with the aortic valve at 12 o'clock and the left atrial appendage at 9 o'clock. PVL formation occurs most commonly anteriorly in the aorto-mitral curtain and along the posterior wall (5–6 o'clock) (14). Since percutaneous PVL closure is currently performed via off-label use of septal or ductal occluder and vascular plug devices, proper device sizing for the crescentic or irregular defect can be challenging. Oversizing is necessary to completely occlude the PVL and to minimize the risk of device embolization. However, excessive protrusion of the occluder disks inwards of the sewing ring introduces the risk of interference with valve leaflet motion, which is particularly harmful in mechanical valves. Therefore, oblong defects may be preferentially closed with multiple small occluder devices with smaller disks than a single large device, particularly if the defect is transected by partially dehisced sutures, which may not allow full expansion of a single large device. Due to these procedural implications, precise imaging for characterizing PVL is essential. Both 3D TEE and CCTA allow for visualization of the size and shape of the paravalvular defect and its spatial relationship to surrounding structures. Furthermore, PVL severity can be quantified by Doppler techniques on TEE. Since dropout artifact can often overestimate the size of the PVL on 3D TEE, it is generally recommended to size and grade severity of the defect with color Doppler (Figure 2).

### Role of TMVR in Mitral Annular Calcification

Transcatheter repair with edge-to-edge (e.g., MitraClip™) or annuloplasty techniques may not be feasible in the presence of significant MAC. Transcatheter replacement (ViMAC TMVR) can be accomplished with a balloon-expandable aortic valve in the mitral position with MAC serving as an anchor. High technical success of ViMAC TMVR has been demonstrated in the TMVR in MAC Global Registry (15) and the TMVR Multicenter Registry (2). However, higher rates of LVOT obstruction (LVOTo) than ViV and ViR TMVR, as frequent as 39.7%, have also been observed and correlate with mortality. Careful pre-procedural planning with CT imaging plays a crucial role in reducing the risk of LVOTo and other complications, as will be discussed in subsequent sections.

### Anatomic Considerations for TMVR

A key component of pre-procedural imaging for TMVR is optimal sizing of the transcatheter valve (THV) for the existing surgical bioprosthesis, annuloplasty ring, or native annulus. Although not yet commercially available, a wide range of dedicated TMVR devices for the native mitral annulus with various fixation mechanisms are currently under clinical investigation. Specific anatomic considerations for each investigational device is beyond the scope of this article.

Due to the presence of an existing surgical bioprosthesis that provides anchoring at the landing zone, sizing for ViV TMVR is more straightforward than for native valve TMVR. Based on the internal diameter of the surgical prosthesis, per manufacturer specifications, THV size is typically selected to allow for up to 10% oversizing for sufficient anchoring and minimize PVL (16). More aggressive oversizing may risk under-expansion of the THV, resulting in unfavorable valve hemodynamics and potential for accelerated SVD (17). The Valve In Valve Mitral mobile app has been developed to aid THV selection for ViV and ViR TMVR (18). However, since degenerated surgical prostheses may have variable degrees of leaflet thickening, calcification, pannus, and annular deformation, CCTA, and 3D TEE imaging provide additional granularity to TMVR sizing. Furthermore, CCTA is necessary for predicting the risk of LVOTo in the consideration for TMVR. In general, CCTA for TMVR planning is performed with arterial-phase contrast, complete cardiac coverage, including the LV apex, and multiphase acquisition throughout the entire cardiac cycle (19). Multiplanar reconstruction with post-processing software allows for the measurement of the actual inner diameter of the surgical prosthesis.

Mitral annuloplasty rings vary in rigidity and circumferential coverage, which affect ViR TMVR planning. Incomplete rings, or bands, are C-shaped and typically anchored to the posterior mitral annulus. Complete rings generally assume a D-shaped configuration and can be rigid, semi-rigid, or flexible (also termed complete bands). Although strategies are often personalized by the cardiothoracic surgeon, complete rigid rings are more often used for functional MR, whereas flexible or semi-rigid rings are more often used for degenerative MR (20). Implantation of a cylindrical THV into a rigid D-shaped ring may result in paravalvular regurgitation due to inadequate sealing. Therefore, flexible or semi-rigid annuloplasty devices may allow for more favorable TMVR conformation. However, the more flexible rings or bands may have a higher risk of LVOTo by allowing the THV to push the anterior mitral leaflet into the outflow tract (21).

**Figure 3:**
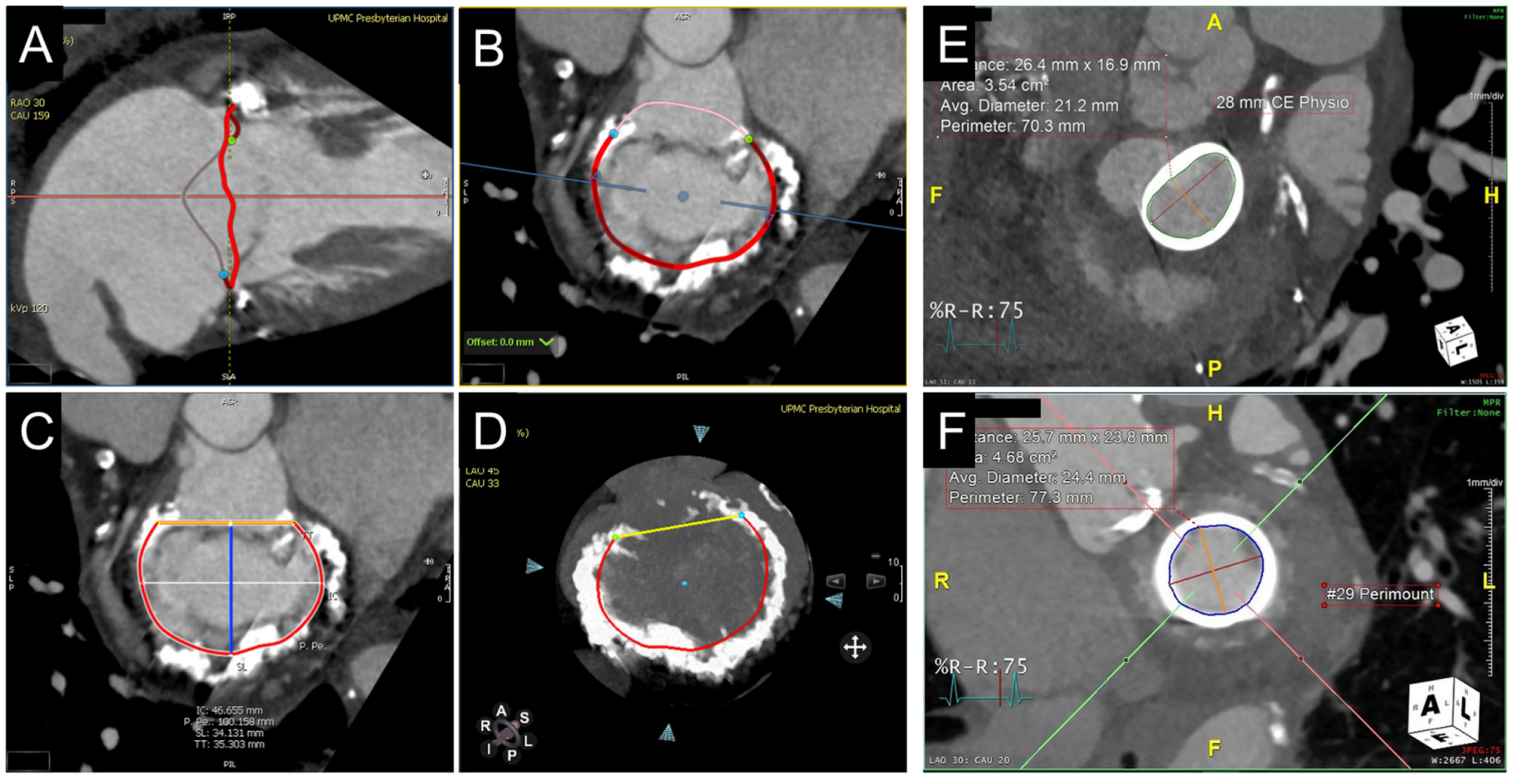
CT annular segmentation for TMVR. **(A–D)** Segmentation of the native mitral annulus for the planning of valve-in-MAC TMVR. As shown from a long-axis view **(A)**, the mitral annulus outlined in red **(A)** has a non-planar saddle geometry. Projected into a short-axis view **(B)**, the anterior horn (*pink border*) may be truncated to assume a D-shaped configuration. **(C)** The trigone-trigone (*orange*), septal-lateral (*blue*), and inter-commissural (*white*) distances are measured from this view. Maximum intensity projection **(D)** allows for assessment of the distribution and density of annular calcification. **(E)** Valve-in-ring TMVR measurements of a complete semi-rigid ring with the major diameter in the inter-commissural dimension (*red*) and the minor diameter in the septal-lateral dimension (*orange*). **(F)** Valve-in-valve TMVR assessment showing the internal diameter slightly under the manufacturer specifications for the bioprosthesis.

The native mitral annulus is more geometrically complex with greater anatomic variability and frequently asymmetric distribution of calcium, which complicates the assessment for ViMAC TMVR. Although 3D TEE allows for segmentation of the saddle-shaped annulus, CCTA remains the mainstay due to its higher spatial resolution. Approximation of the saddle-shaped annulus using a cubic spline interpolation method, with measurements projected onto a flat plane to yield a D-shaped contour, has been the most commonly used method to date (22). When using a similar method, 3D-TEE agrees and correlates well with CCTA and can be used as an alternative imaging strategy when CCTA is not feasible (23). Dimensions measured at the landing zone include the trigone-to-trigone (TT), septal-lateral (SL), and intercommissural (IC) distances, as well as the annular area. Of note, the SL distance corresponds to the anterior-posterior minor diameter of the annulus, measured at A2-P2 on echocardiography. The major diameter generally corresponds to the IC distance. Given the dynamism of the mitral annulus, measurements are ideally performed in diastolic and systolic phases. Dense or caseous MAC may affect the accuracy of measurements at the landing zone and carry other implications complicating TMVR sizing. Characterization of and grading systems for MAC are evolving (24). While MAC serves as an anchor for TMVR implantation, its irregularity and rigidity may lead to inadequate seal and subsequent paravalvular leak. Asymmetric distribution of MAC in the posterior annulus may also influence the risk of LVOTo (Figure 3).

### Prediction of LVOT Obstruction

As discussed previously, LVOTo is a serious complication of TMVR, more common in ViMAC cases. The incidence of LVOTo, defined as an increase in the LVOT gradient ≥10 mmHg from baseline, is estimated at 7.1% in the TMVR Multicenter Registry (7) though the rate of hemodynamically significant LVOTo may be lower (25). Implantation of a THV in a native mitral valve displaces the anterior mitral leaflet (AML) septally and extends the effective outflow tract ventricularly, creating a neo-LVOT (26). Similar to hypertrophic obstructive cardiomyopathy, narrowing in the neo-LVOT and systolic anterior motion of the AML due to Venturi forces can result in dynamic LVOTo. In ViV TMVR, the frame and open leaflets of the bioprosthetic valve form the boundary of the neo-LVOT rather than the more mobile native AML. Therefore, dynamic LVOTo is less likely in ViV cases, though fixed obstruction is possible. Anatomic factors that can contribute to LVOTo with TMVR include a small predicted neo-LVOT area, basal septal hypertrophy, AML elongation, small LV cavity, and acute aortomitral angle (26). Device-related factors that influence LVOTo include the depth of THV implantation, THV size (particularly height), and distal flaring of the THV. Finally, LVOTo is also dependent upon hemodynamic loading conditions and LV contractility.

**Figure 4:**
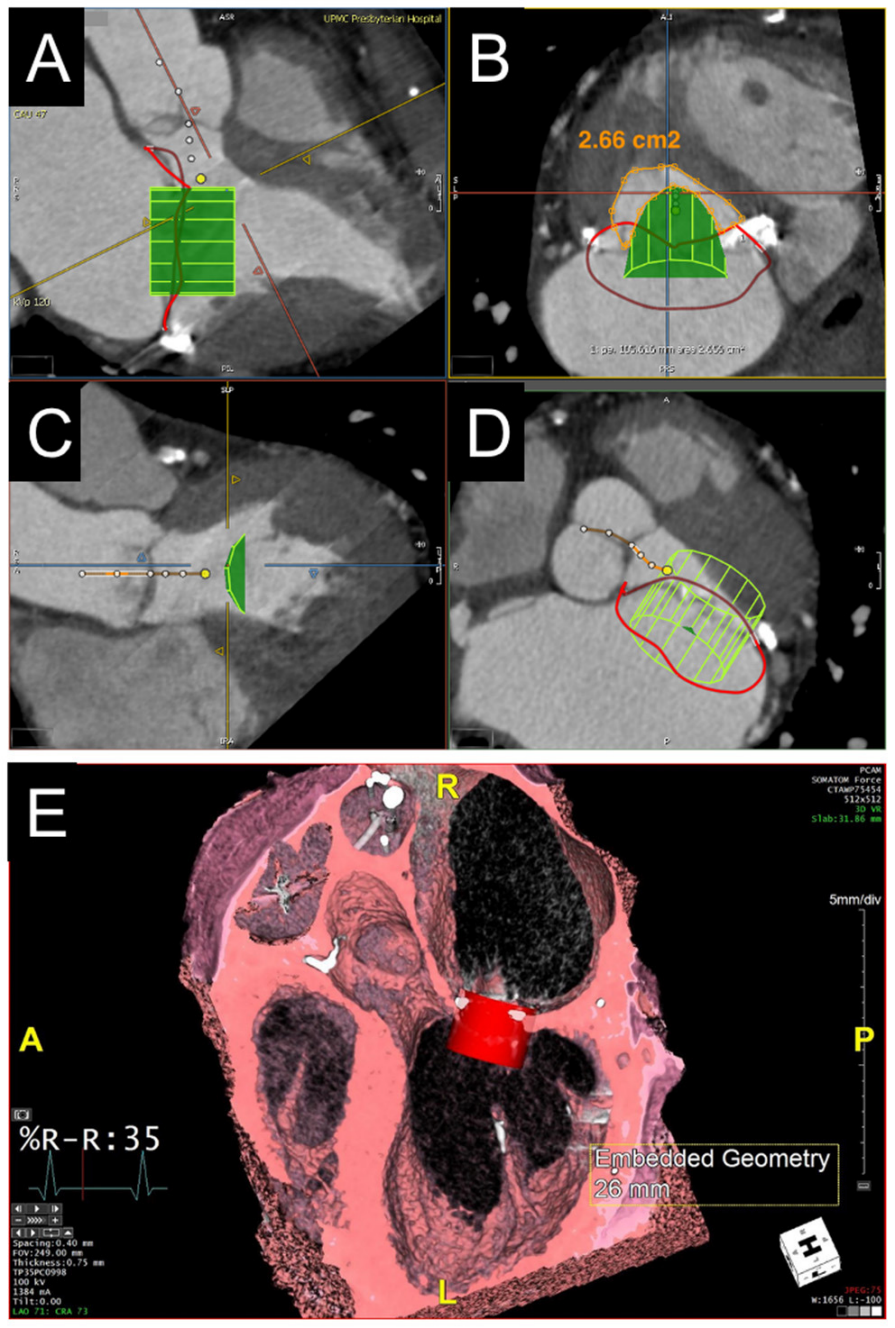
Evaluation of predicted neo-LVOT area by CT. **(A–D)** LVOT assessment for valve-in-MAC TMVR. Virtual implantation of a cylindrical transcatheter valve coaxial to the mitral annulus during a systolic phase **(A)** and delineation of the LVOT centerline yields a short-axis view of the predicted neo-LVOT area (**B**, *orange*), which measures 2.66 cm^2^ and predicts a low risk of LVOT obstruction with TMVR in this case. **(E)** Volume rendered image showing a capacious neo-LVOT area in a case under consideration for valve-in-ring TMVR.

Prediction of LVOTo risk by CCTA imaging is a crucial aspect of the pre-procedural assessment for TMVR. Echocardiography can be utilized to establish baseline LVOT gradients and qualitatively assessing for anatomic factors, such as LV size and septal thickness and contour. Nonetheless, CCTA remains the primary imaging modality for annular segmentation and neo-LVOT prediction. TMVR simulation with THV models on post-processing software allows for direct planimetry of the area of the predicted neo-LVOT (Figure 4). Depth of implantation can vary, but generally, ~80% of the THV is ventricular and 20% atrial to ensure stability. Although many early investigators recommended measurement of the neo-LVOT area at end-systole, current research indicates early-mid systolic measurements better predict post-implant LVOTo (27). A measured neo-LVOT area of <170 mm^2^ has been shown to predict a significant risk of LVOTo with TMVR (28), whereas a relative reduction of ~60% of the LVOT area has been suggested by other investigators (29). By facilitating a more interactive representation of patient-specific anatomy and neo-LVOT, 3D printed models derived from CCTA have also been used for TMVR planning (30) and can also be done so with 3D TEE or CMR datasets (albeit with lower spatial resolution). Notably, dynamic factors such as volume status and loading conditions are not accounted for by planning with either CCTA or 3D printing. As will be discussed in later sections, intentional laceration (LAMPOON procedure) of the AML, if non-calcified, may increase the neo-LVOT area and avoid obstruction (31, 32). Alternatively, perfusion balloon inflation in the LVOT has also been described as a technique to maintain patency of the LVOT during TMVR deployment (33, 34). Alcohol septal ablation can increase the CCTA-predicted neo-LVOT if done pre-emptively or reduce LVOT gradients as a bailout option intraprocedurally (35).

### Percutaneous Access Planning

Percutaneous access for TMVR is generally accomplished with a transapical (60%) or a transseptal (40%) approach, and a direct open transatrial approach (1%) is performed much less often, based on the TMVR Multicenter Registry (2). For the transapical approach, CT imaging can help with planning of the optimal access site. In order to ensure coaxiality of TMVR device deployment and perpendicularity with the mitral annular plane, the epicardial puncture site is typically anterolateral to the true LV apex. The entire proposed trajectory can be assessed by CCTA for its proximity to coronary arteries, scarred myocardium, papillary muscles, and any surrounding extracardiac structures. Extending the transapical trajectory to the chest wall virtually on CT also allows for the identification of the optimal intercostal space and distance of the puncture site from the sternum.

While the transapical approach allows for a more straightforward trajectory for coaxial TMVR deployment, a transseptal transvenous approach avoids the need to perforate the apical myocardium, creating scar, and may be associated with improved outcomes (36, 37). Transvenous access for TMVR is typically achieved through the femoral vein. CCTA planning should therefore involve surveillance of the peripheral vasculature with a venous phase scan. Similar to arterial evaluation for transcatheter aortic valve replacement, the venous vasculature is evaluated for obstruction, caliber, and tortuosity. For the transseptal puncture, planning with CT can help optimize the puncture site in the fossa ovalis. To accomplish this, the CCTA protocol should yield some right-sided contrast opacification. As will be further discussed later, the atrial septal anatomy can be evaluated by TEE for features that could make the transseptal puncture more challenging, including lipomatous hypertrophy, atrial septal aneurysm, or a patent foramen ovale.

**Figure 5:**
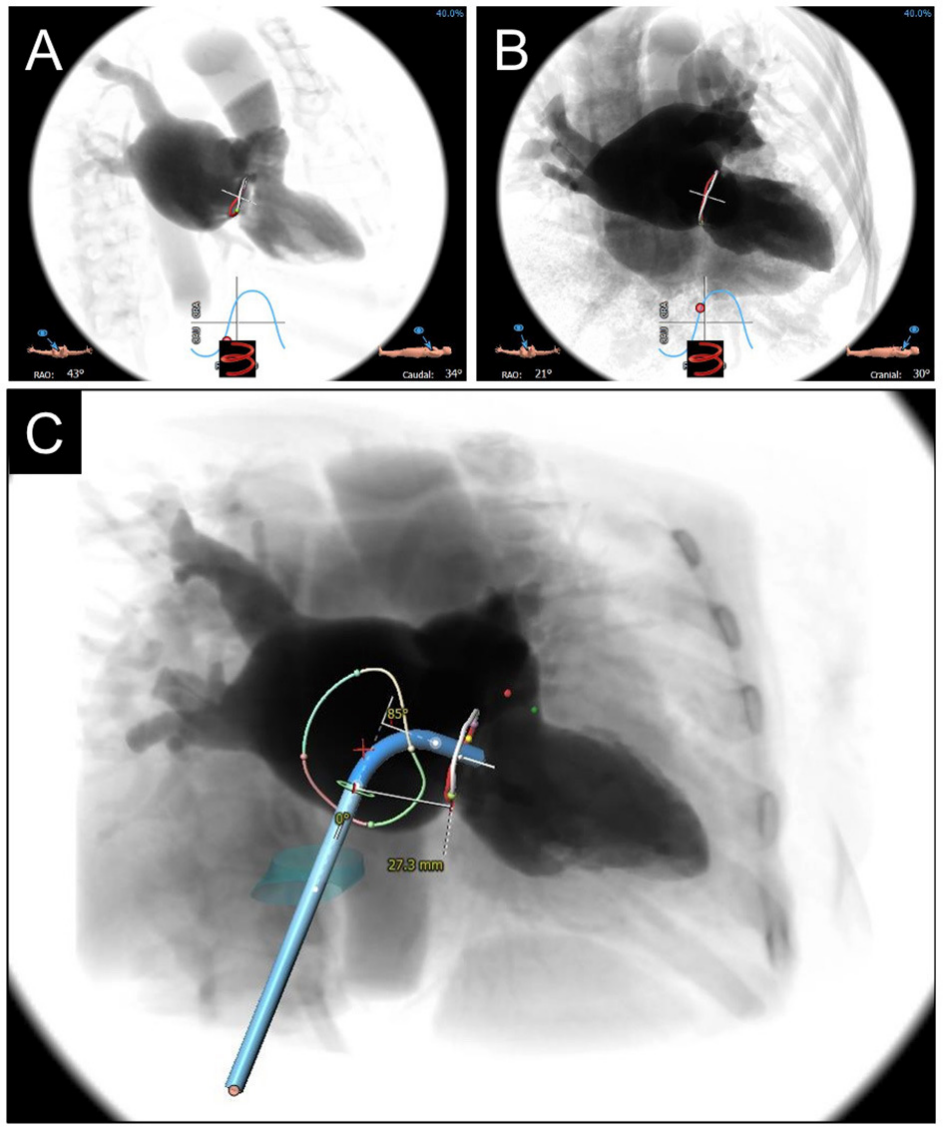
CCTA-based Fluoroscopic Simulation for TMVR access planning. Projected Fluoroscopic coplanar angles can be found along the displayed S-curve. A coplanar working view **(A)** and modified 4-chamber view **(B)** are displayed for a planned valve-in-ring procedure. **(C)** Full transseptal access planning is shown, with a projected catheter path through the IVC (turquoise cylinder) and across the interatrial septum for coaxial valve implantation from the working view. Catheter bend angle is displayed (85 degrees) and ideal entry distance from the mitral annulus (27.3 mm).

Fluoroscopic angles predicted by CT imaging can streamline TMVR deployment at the time of the procedure (Figure 5). After segmentation of the mitral annulus by CT, coplanar fluoroscopic projections can be formulated along an S-shaped curve with corresponding angles in the left anterior oblique (LAO) or right anterior oblique (RAO) direction and cranial-caudal dimension. Using the fluoroscopic angles along this curve that create coplanar TT (commissural) and SL (3-chamber) views can help in achieving coaxiality at the time of TMVR deployment. Of note, C-arm angulations for the coplanar TT view may not be practical, and a slightly compromised view may be utilized instead. For planning transapical access, the fluoroscopic angle (LAO caudal) for the short axis *en face* view of the mitral annulus can also be determined by CCTA.

## Intraprocedural Guidance

Transcatheter mitral valve interventions should be performed in a standard cath lab with adequate equipment, allowing for the simultaneous display of fluoroscopy and echocardiographic images. Ideally, this should include fusion imaging of fluoroscopy with computed tomography datasets and/or real-time echocardiographic imaging, which can increase procedural success and decrease procedure duration and radiation dose (38, 39). Currently, TEE is the gold standard for intraprocedural imaging for these interventions, which are therefore performed under general anesthesia.

Immediately after induction of general anesthesia, a baseline TEE is performed to confirm the previous findings in terms of severity of mitral valve pathology, LV function, and wall motion; determine baseline trans-mitral velocity and gradients, pulmonary vein flow velocity, and direction; and exclude significant LVOTo, left atrial appendage thrombus, and pericardial effusion. With the use of 3D imaging, a surgeon's view can be obtained to characterize the mitral valve structure and location of pathology. Other cardiac structures and valves should also be assessed to ensure there have been no significant changes from prior imaging. Any new findings should be correlated with prior imaging accounting for the different hemodynamic conditions that exist under general anesthesia and discussed with the interventional team prior to the initiation of the procedure.

### TMVR

#### Transapical Access

When a transapical approach is utilized (such as for most investigational devices for TMVR in native mitral annulus), TEE imaging can assist in localizing the ideal site of apical incision for coaxial device delivery into the mitral valve annulus (40, 41). After performing a lateral thoracotomy and exposing the LV apex, the surgeon will apply pressure with their finger under direct TEE visualization to determine the best site for an incision. This ideal location can also be facilitated by pre-procedural CCTA analysis, as previously discussed, and fusion of CT with fluoroscopy (42). Percutaneous transapical access and closure have also been utilized in selected cases (43, 44). The THV is then advanced over a guidewire toward the mitral annulus or existing bioprosthesis and then deployed under live echocardiographic guidance. At the end of the procedure, the surgeon will achieve hemostasis and surgical closure of the defect and thoracotomy incision.

#### Transseptal Access

**Figure 6:**
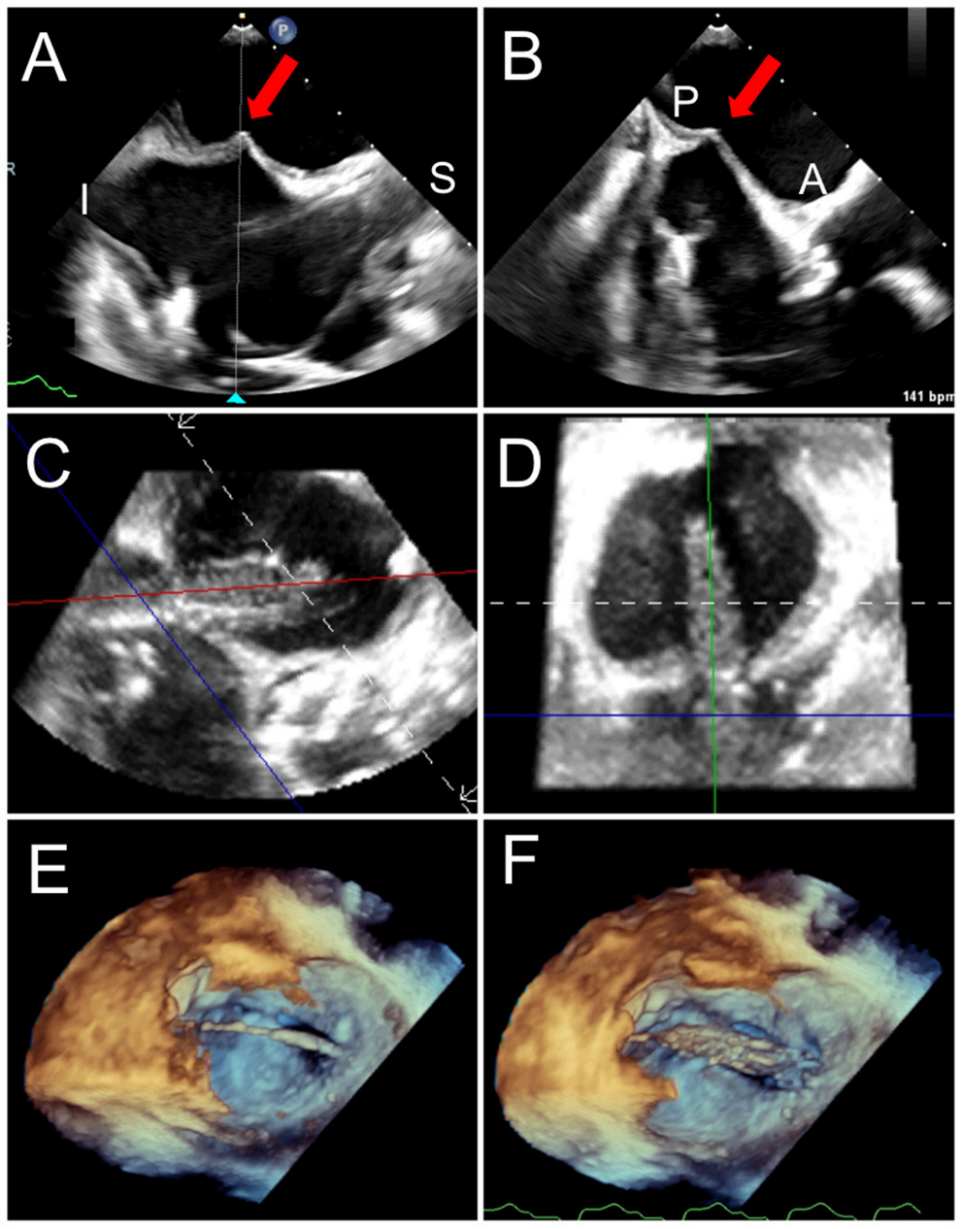
Trans-septal puncture and septostomy for TMVR. A transseptal needle is seen tenting the interatrial septum (red arrows), best assessed utilizing biplane TEE imaging in the bi-caval (superior-inferior, **A**) and short-axis (anterior-posterior, **B**) views to confirm positioning in the inferior and posterior aspect of the fossa ovalis. After a successful transseptal puncture, a balloon is advanced over a stiff wire, and proper positioning across the septum can be verified utilizing live 3D MPR **(C,D)**. **(E)** The balloon is visualized in a 3D zoom volume-rendered image from the left atrial perspective. **(F)** The balloon is inflated for atrial septostomy in order to later accommodate the large crimped TMVR device. Supplementary Video 1 illustrates balloon inflation for atrial septostomy, as seen from the left atrium.

The ideal and less invasive approach for TMVR is via transseptal puncture; in addition to being preferred by patients, it may allow for improved LV function and lower complications as compared to a transapical approach (37, 45). The transseptal approach, though technically more challenging, can be used for the balloon-expandable Sapien 3 valves (Edwards Lifesciences, Irvine, CA) for ViV, ViR, and ViMAC, as well as the investigational devices for valve in native annulus implantation, including CardiAQ-EVOQUE and Valtech CardioValve (Edwards Lifesciences, Irvine, CA), Cephea (Abbott Structural, Santa Clara, CA) and HighLife (HighLife Medical, Irvine CA) (36, 37, 46–49). After venous and arterial access is obtained, the interventionalist advances a transseptal needle and sheath over the wire into the superior vena cava, then slowly withdraws it into the right atrium under TEE guidance, until tenting of the septum is noted. The needle position is assessed in the mid-esophageal bi-caval view (superior-inferior) and short-axis view (anterior-posterior), and height can be confirmed in a 4-chamber view. Transseptal puncture for TMVR is usually performed in the infero-posterior portion of the fossa ovalis, or the ideal position as determined by pre-procedural CCTA analysis, which may be projected to real-time TEE via fusion imaging (50). Orthogonal bi-plane imaging and live 3D imaging can simultaneously display all four rims of the fossa ovalis and serve for confirmation (Figures 6A,B). Fusion of real-time TEE and fluoroscopy can further facilitate transseptal sheath positioning. Once the ideal puncture site is achieved, the needle and sheath will be advanced into the LA under continuous TEE visualization. The transseptal sheath is subsequently exchanged by the specific device sheath. A pigtail catheter is advanced over a guidewire across the mitral valve toward the LV apex and subsequently exchanged by a curved stiff wire. Due to the larger dimensions of the TMVR valves and delivery system (as compared with edge-to-edge repair), a balloon atrial septostomy is necessary and performed under TEE and fluoroscopic guidance (Figures 6C–F, Supplementary Video 1).

#### Device Positioning and Deployment

**Figure 7:**
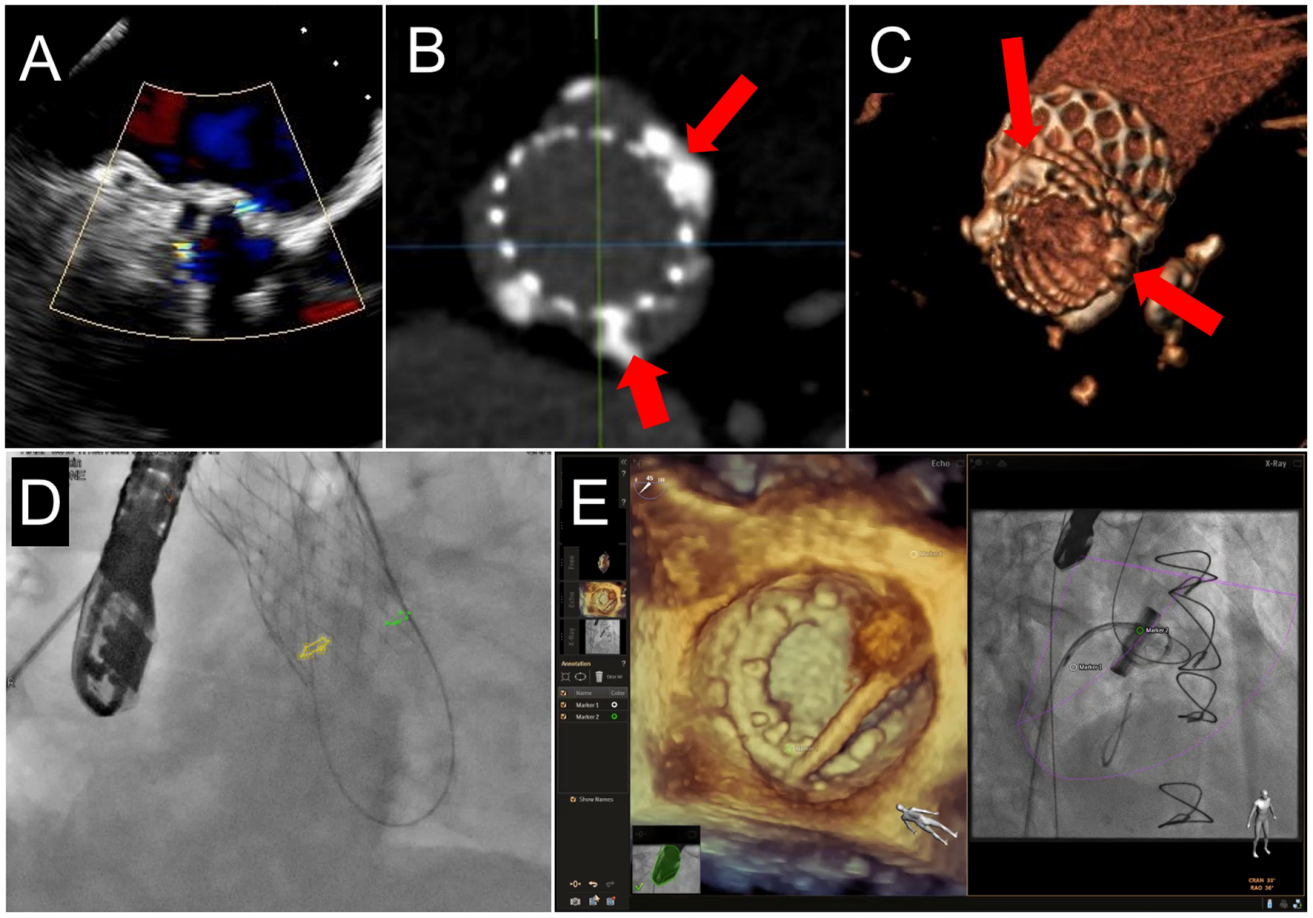
Fusion Imaging. 2 PVL jets are seen on TEE color Doppler imaging in a patient who had previously undergoing TAVR **(A)**. These were anatomically located on subsequent CCTA (**B,C**, red arrows) and co-registered onto fluoroscopic procedural imaging (**D**, yellow and green circles). **(E)** A mitral PVL closure using live TEE fusion imaging for co-localization.

Position is key in device stability and reducing the risk of LVOTo. Positioning the THV too apically can compromise LVOT flow, whereas deploying too atrially can compromise device stability and risk embolization. For ViR or ViMAC utilizing a balloon-expandable Sapien 3 THV, it is generally desirable to have ~80% of the valve stent in the LV and 20% in the LA. During deployment, the balloon-expandable THV shortens almost exclusively from the inflow (atrial) side; therefore, the landing zone is assessed based on the ventricular edge of the stent. For ViV TMVR, it is generally recommended to align the ventricular edge of the THV frame with the ventricular edge of the existing bioprosthesis, which can be assessed by fluoroscopic markers and TEE, but this positioning can be altered based on the risks of LVOTo vs. atrial embolization, as well as the intended degree of valve expansion (37, 45, 47). In mitral bioprostheses and rings with few fluoroscopic markers, TEE fusion can help optimize the depth of TMVR implantation (Figures 7D,E). Positioning of the investigational valves for TMVR in a native mitral annulus will depend on the specific device design and morphology. Anchoring mechanisms for existing devices include ventricular tabs, radial force, atrial and ventricular disks, annular or subvalvular docks, among others. The full discussion of investigational devices for TMVR in native mitral annulus is beyond the scope of this review. To optimize visualization of the device trajectory and coaxiality, simultaneous biplane imaging is performed from the mid-esophageal commissural view (40–70°) to assess the medial-lateral position and the mid-esophageal long-axis view (120–150°) to assess the anterior-posterior position. Live 3D multiplanar reconstruction (MPR) allows for more accurate alignment of the echocardiographic planes with the device and delivery system during device positioning and deployment. However, 3D TEE reduces frame rate, which may be overcome by decreasing the sector size, adjusting the spatial resolution, and utilizing multi-beat acquisition.

**Figure 8:**
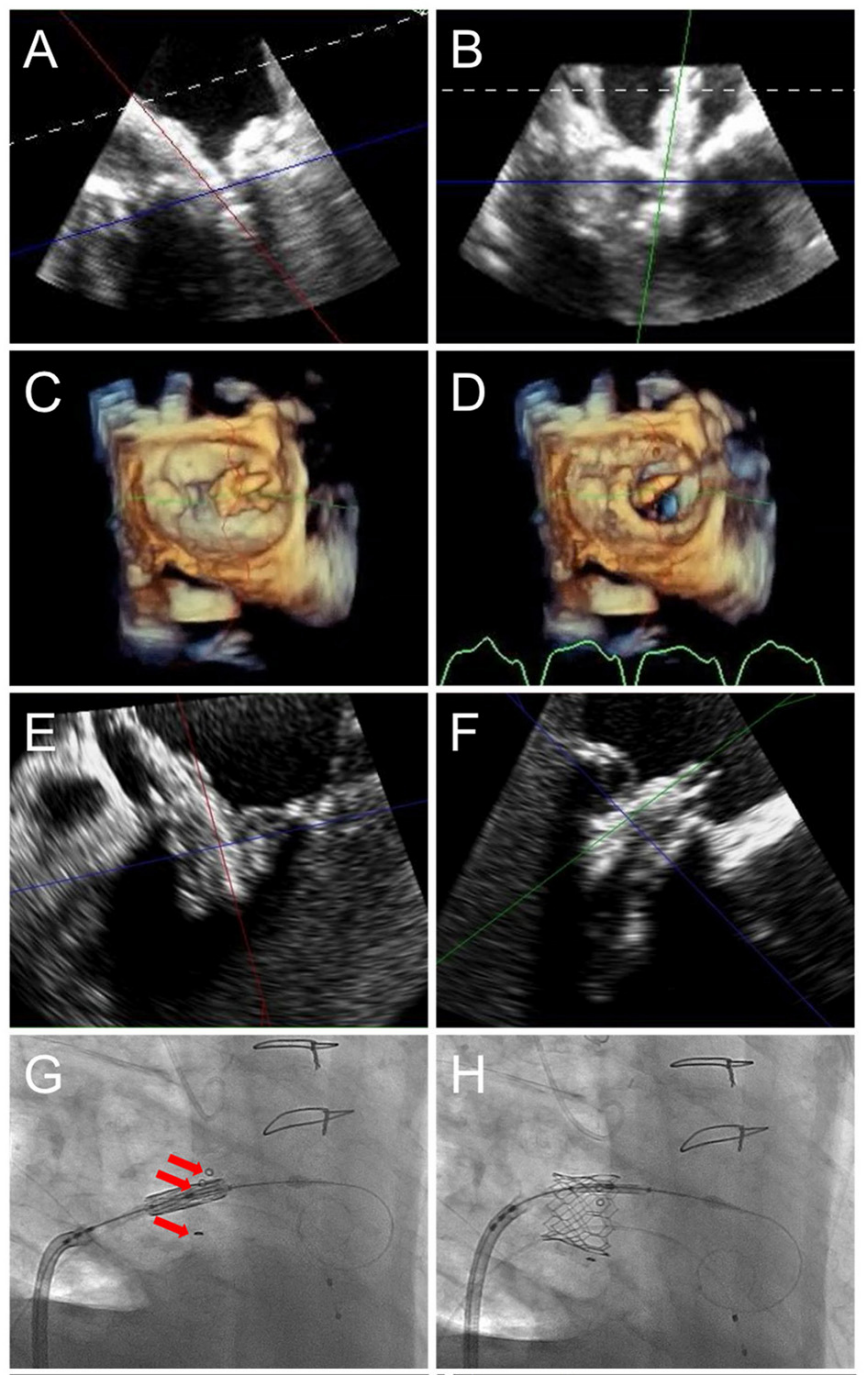
TMVR device positioning. As the orientation of the device may differ from that of the ultrasound plane, live TEE 3D MPR is helpful to properly assess positioning in regards to a calcified mitral annulus (ViMAC) or prior surgical ring (ViR) **(A,B)**. Valve deployment can also be visualized in an en-face surgeon's view (**C,D**, see Supplementary Video 2). In the case of a valve in valve implantation, a 26 mm Sapien S3 valve is seen being positioned inside a 29 mm bioprosthetic surgical valve in 3D MPR **(E,F)**, confirming alignment of the ventricular edge of the THV valve frame with the ventricular edge of the existing bioprosthesis which can be identified in fluoroscopy (**G**, red arrows). During deployment **(H)**, the THV shortens predominantly from the inflow (atrial) side, leading to its final position. Supplementary Video 2 demonstrates THV deployment in live 3D MPR.

**Figure 9:**
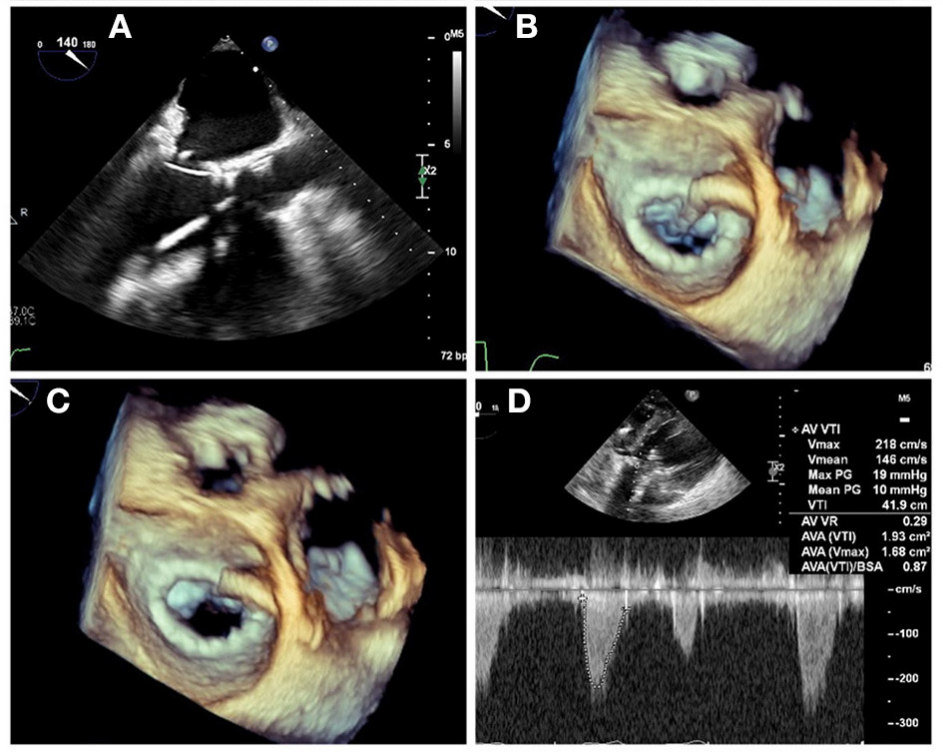
LAMPOON procedure during TMVR. In cases of anticipated LVOT obstruction, intentional laceration of the anterior mitral valve leaflet (LAMPOON procedure) can be performed by connecting a retrograde wire through the aortic valve and an antegrade transseptal wire from the LA to form a loop via snaring **(A)**. The loop is positioned in the trajectory to transect the A2 scallop **(B)**, and tension is applied during radiofrequency energy application to lacerate the anterior leaflet from base to tip **(C)**. In this case, post-valve-in-ring implantation measurement demonstrated a mean gradient across the LVOT of 10 mm Hg **(D)**.

Device deployment, whether self-expanding or balloon-expandable, is performed slowly during rapid ventricular pacing (around 160 bpm) and ventilator hold, under direct TEE visualization (Figure 8, Supplementary Video 2). In selected cases of TMVR in a native valve (including ViMAC and ViR) where a long anterior mitral leaflet is predicted to significantly increase the risk for dynamic LVOTo, a novel technique of intentional laceration of the anterior mitral valve leaflet to prevent outflow obstruction (LAMPOON) can be utilized prior to device implantation (32, 51). The procedure is performed under direct TEE visualization via a retrograde approach through the aorta by transecting the base of the anterior mitral leaflet with one guidewire, which is then snared and pulled outward toward the leaflet tip (Figure 9).

#### Post-deployment Assessment

**Figure 10:**
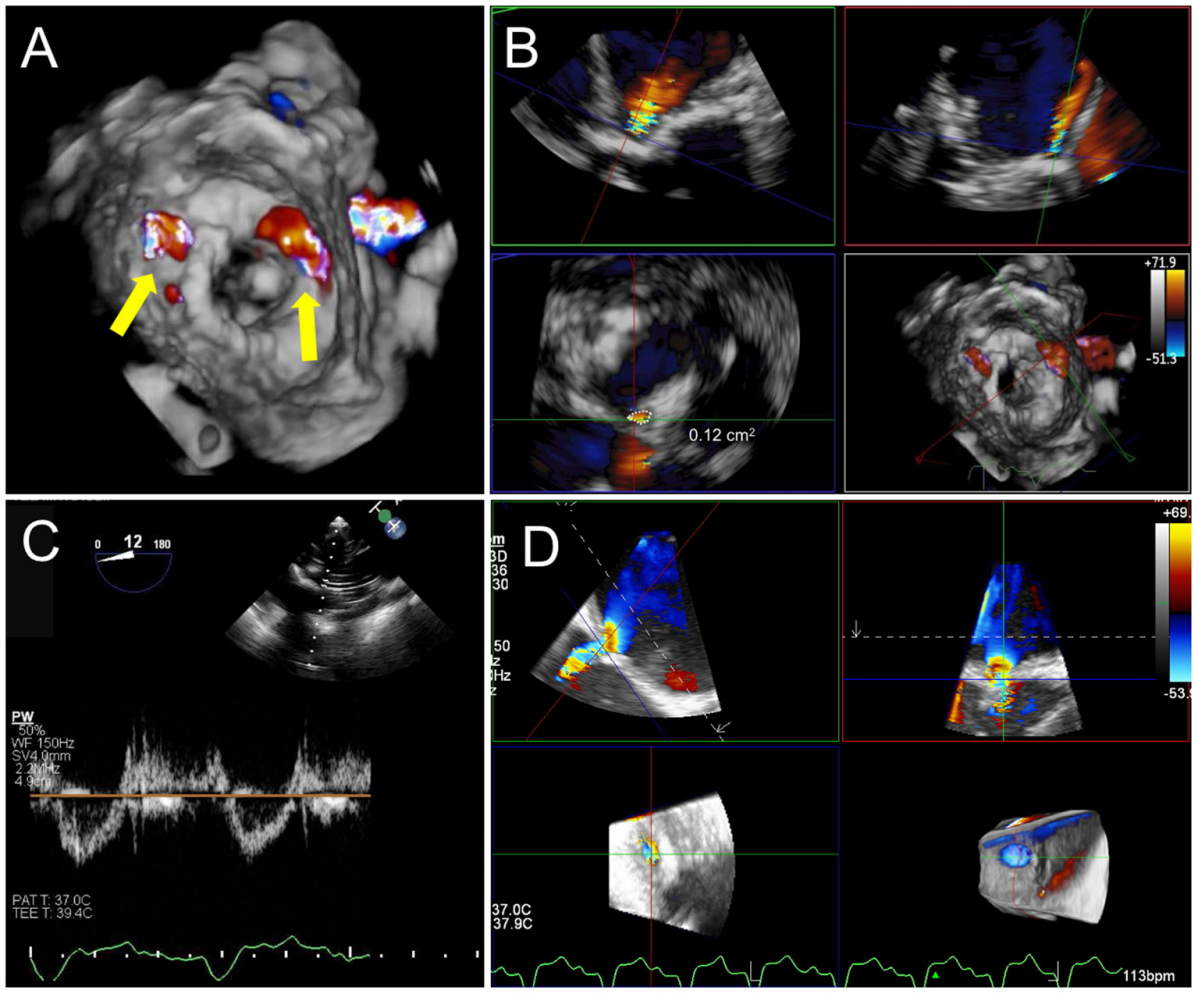
Post TMVR assessment immediately after deployment. **(A)** 3D color Doppler evaluation from an en-face surgeon's view immediately after a valve in MAC implantation reveals two areas of paravalvular leak (PVL) at 10 and 1 o'clock (yellow arrows). See Supplementary Video 3 for 3D en face evaluation of valve morphology and leaflet motion. **(B)** Careful interrogation of the largest area of PVL with 3D MPR reveals a vena contracta area of 0.12 cm2. **(C)** Pulsed-wave Doppler interrogation of the LVOT from a transgastric view in order to exclude a significant gradient or obstruction. **(D)** Evaluation of residual iatrogenic septal defect size and direction of flow.

Immediately after device delivery, a careful TEE evaluation should be performed, aimed at assessing the device position and morphology, hemodynamic changes, presence of valvular or paravalvular regurgitation, LVOTo, and excluding complications such as new pericardial effusion or wall motion abnormalities (Figure 10). Attention should be given to the lateral wall of the LV, atrioventricular groove, circumflex artery, and coronary sinus since trauma and rupture can occur as a result of device deployment and should be promptly recognized. The new valve should be well-seated in the intended position, without evidence for rocking motion (Supplementary Video 3). The valve leaflets should appear pliable with normal motion and laminar flow by Doppler evaluation. Trans-mitral gradients should be again assessed and compared with baseline. PVL can occur when the THV frame is not perfectly apposed against the native annulus, ring, or existing bioprosthesis and should be carefully assessed by 2D and 3D color Doppler interrogation, as previously discussed. As PVL jets can often be multiple and eccentric, 3D color Doppler evaluation is very helpful in the characterization of jet location and direction, as well as quantification by measurement of the vena contracta area in 3D MPR, which requires proper technique ensuring adequate spatial and temporal resolution. Additionally, the pulmonary vein flow should be assessed bilaterally, and systolic antegrade flow should be confirmed. Moderate or greater degree of PVL is associated with adverse outcomes and may require repeat balloon inflation or percutaneous closure with an occluder device prior to finalizing the procedure.

Given the significant risk for LVOTo, particularly with a valve in a native annulus, it is imperative to carefully assess LVOT flow and gradients after device deployment. In addition to a mid-esophageal long-axis view for color Doppler assessment, evaluation of LVOT gradients is best achieved by PW Doppler interrogation from a deep trans-gastric view. MPR of a high-quality 3D TEE dataset can be utilized to measure the resultant neo-LVOT area. However, acoustic shadowing is a limitation of TEE visualization, and there may be a role for TEE-CT fusion for neo-LVOT assessment (52).

Finally, after device deployment and following removal of the delivery system and sheath, attention should be paid to the interatrial septum to identify and assess the iatrogenic atrial septal defect (iASD) and direction of flow. Though many of these defects resolve spontaneously and are without clinical consequences, persistent iASD at 6 months has been associated with adverse outcomes after transcatheter mitral valve repair (53). The immediate closure of iASD may be necessary if hypoxia and a right-to-left shunt are noted or if the defect is very large.

### PVL Closure

#### Transseptal Puncture

**Figure 11:**
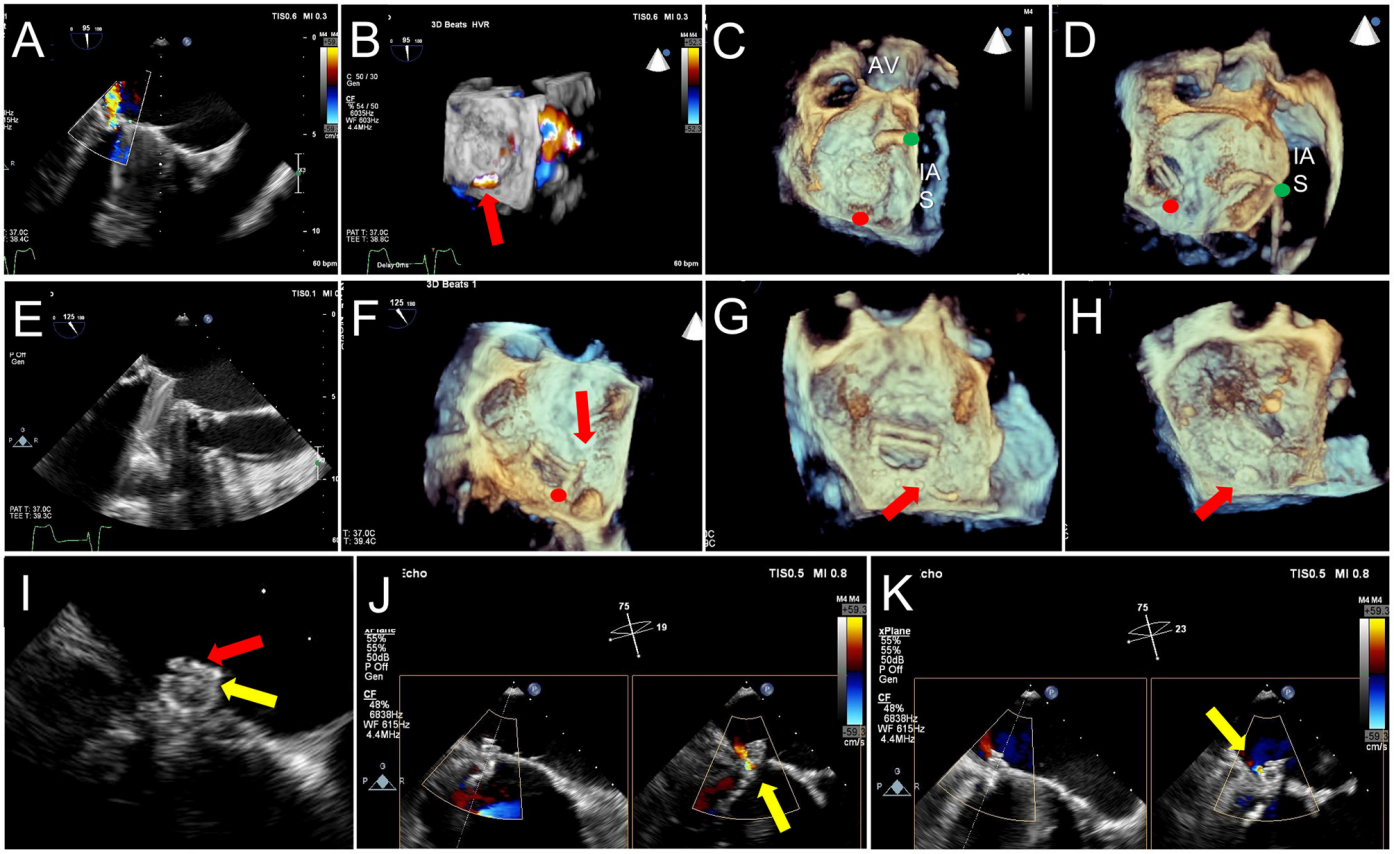
TEE Guidance of Challenging Closure of Posteromedial PVL. A posterior paravalvular leak resulting in hemolysis is visualized by 2D color Doppler **(A)** and 3D color Doppler Imaging (between 5 and 6 o clock in a surgeon's view, red arrow, **B)**. **(C)** The first attempt at transseptal puncture is shown (green circle). The location of PVL is marked by a red circle. **(D)** After unsuccessful attempts to cross the defect, the septum was repunctured more posterior (green circle) for a better trajectory toward the defect. **(E)** After additional unsuccessful attempts, the approach was changed to retrograde. **(F)** As acoustic shadowing limits visualization on the ventricular aspect of the mechanical mitral prosthesis, wire exit point was visualized in the left atrium. Here, the wire is visualized inside the sewing ring (red arrow) adjacent to the PVL (red circle). **(G)** After reorientation of the wire, the defect was successfully crossed (red arrow). **(H)** The atrial disk of an AVPII closure device is visualized after deployment on 3D imaging (red arrow). **(I)** 2D echocardiography demonstrates the atrial disk (red arrow) and a portion of the center cylinder (yellow arrow). **(J)** A residual peri-device jet is seen (yellow arrow). **(K)** After device re-sheathing and slight repositioning, there is a trivial residual PVL jet flowing through the center of the closure device (yellow arrow). AV, aortic valve; IAS, interatrial septum.

The first consideration for intraprocedural guidance is the location of the transseptal puncture. Lateral and anterior jets are the most accessible from multiple puncture sites. Posterior and medial jets are more challenging and require careful consideration of transseptal puncture location on a case-by-case basis (Figures 11A–D). While the general location of the jet may be accessible in a posterior or medial defect, the direction through which to cross the defect will be more complex. A steerable sheath allows significant flexibility; however, for entry into the defect, a catheter with a greater bend may be required to approach the defect with a favorable trajectory. For defects that are difficult to cross via a transseptal route, retrograde access may provide an easier option (Figures 11E–G). Transapical access may also be considered for greater support, with the tradeoff of a more invasive approach. Correlative or fusion imaging with CCTA may help better understand the relationship of the PVL with fluoroscopic views and reduce the procedural time (Figures 7A–D) (54). Care should be taken to avoid transseptal puncture near the interatrial or atrial-caval grooves, to avoid atrial perforation and potential cardiac tamponade.

#### Closure of Defect

Once the guide or sheath is situated across the interatrial septum, 2D and 3D TEE imaging play a crucial role in guidance across the defect. Besides general location, the imager can guide the trajectory of the guide/sheath, crossing wire, and exchange catheter. Rotation of the 3-dimensional surgical view, combined with periodic switching to 2D TEE imaging, should be used to appreciate the angulation of the guiding sheath, catheter, and wire. Appropriate adjustments to sheath direction and catheter type can be made by the interventionalist with input from the imager. Real-time TEE and fluoroscopic fusion imaging can greatly facilitate PVL defect crossing, particularly in rotated hearts or small defects (Figure 7E). Trajectories that are not facing the defect may require banking a wire down the atrial wall and/or catheters with a greater angle or curvature; this is often difficult to appreciate by fluoroscopy alone; hence, imaging guidance is critical to reducing procedural time. Once the closure device is across the defect, an attempt should be made to visualize the ventricular disc of the device as it comes against the prosthetic sewing ring, followed by the center column (if present) and the atrial disc (Figures 11H,I).

#### Post-closure Evaluation

**Figure 12:**
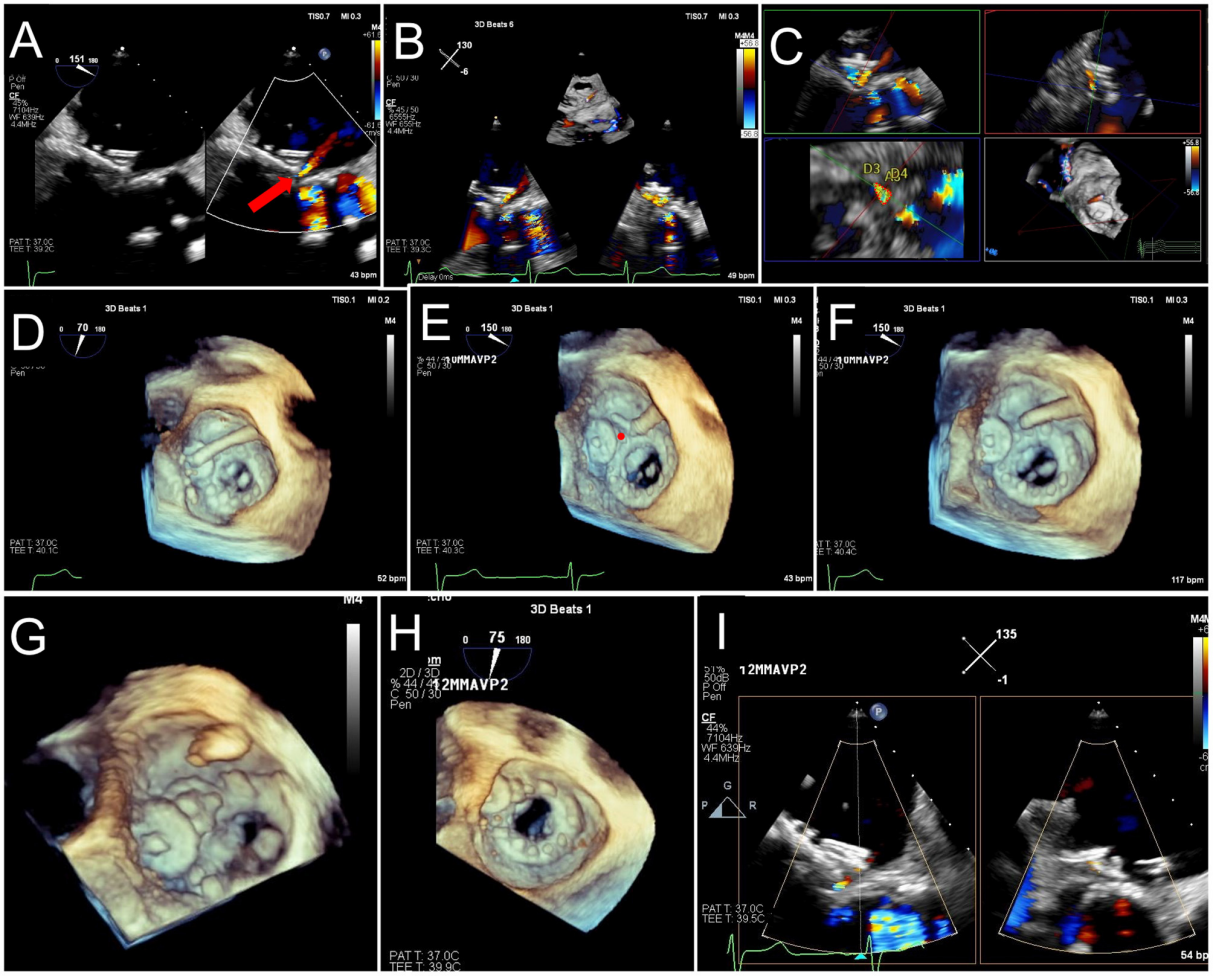
TEE Guided Implantation of a Second PVL Closure Device for Residual Leak. **(A)** After an initial deployment of a 10 mm AVPII device, a residual leak is seen adjacent to the first device (red arrow). **(B)** A sector-based 3D Color Doppler acquisition demonstrates the location of the residual leak medial to the first device. **(C)** Multiplanar reconstruction of the 3D color Doppler acquisition is aligned with the jet for measurement of the paravalvular defect (lower left panel, dotted red tracing), measuring 17 mm^2^, with dimensions 10 x 4 mm. **(D)** The guide catheter is positioned on top of the first device, too lateral to enter the defect. **(E)** The guide is pulled back, and wire crossing of the defect (red circle) is attempted. Wire trajectory is medial and anterior to the defect. **(F)** After correction of wire trajectory, the defect is crossed. **(G)** A 12 mm AVPII device is deployed within the residual defect. **(H)** Bioprosthetic leaflet opening appears normal, without interference from the closure devices. **(I)** Biplane color Doppler imaging demonstrates trivial residual leak after deployment of the 2nd PVL closure device.

**Figure 13:**
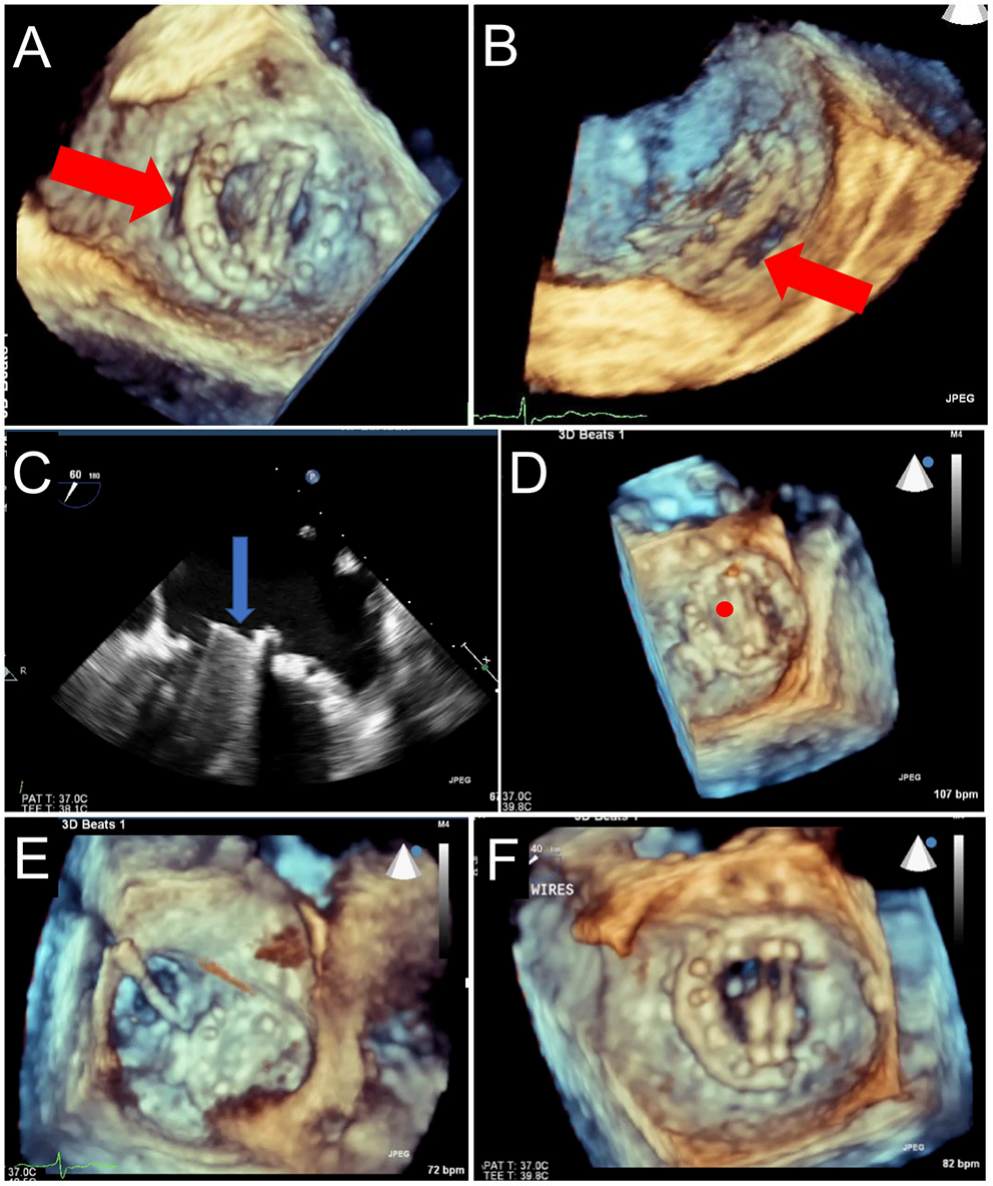
Prosthetic Mechanical Leaflet Dysfunction Following PVL Closure. A large lateral paravalvular defect is seen (red arrows) from the surgical **(A)** and lateral **(B)** view. While initial deployment was successful, once the closure device was released from the delivery system, the lateral mechanical leaflet no longer opened during diastole due to interference from the ventricular aspect of the closure device **(C**, blue arrow, and **D**, red circle). After snaring to remove the closure device **(E)**, leaflet motion returned to normal **(F)**.

Post-closure assessment of paravalvular leak should be performed using 2D and 3D TEE with and without color Doppler and compared to the pre-implant assessment (Figures 11H–K, 12A–C). If results are inadequate, the device could be repositioned within the paravalvular tunnel and imaging re-assessment performed. Prior to device release, one must confirm that the mobility or function of the bioprosthetic leaflet or mechanical prosthetic disc is unchanged. Once the device is released, it may change position when the torque of the delivery system is removed; thus, re-assessment of regurgitant severity and leaflet/disc function is required (Figure 13). If further closure devices are to be placed adjacent to the first, re-assessment of regurgitant severity and device positions must be performed after each device is deployed, and wires guided into the appropriate locations. Once the guidance equipment is removed from the septum, imaging assessment for pericardial effusion should be performed.

## Evolving Imaging Technologies

### Advances in Intracardiac Echocardiography and Fusion Imaging

Although intracardiac echocardiography (ICE) procedural guidance has been well-described in the setting of left atrial appendage occlusion (55, 56) and to a lesser degree in mitral balloon valvuloplasty and transcatheter aortic valve replacement (57), there are limited reports of ICE-guided TMVR or PVL closure (50). ICE from the right atrium is particularly limited in visualizing lateral mitral PVL defects, and ICE in the LA requires an additional transseptal puncture and its associated risk. Development of multiplanar and 3D ICE may increase its utilization for TMVR and PVL closure guidance, particularly if it obviates the need for a dedicated imager. However, cost and operator experience may remain important limitations of ICE. Fusion of TEE with fluoroscopy is currently primarily limited by inter-vendor compatibility. While CT co-registration does not have this limitation, it is hindered by the need for specialized software and non-dynamic visualization. Development of vendor-neutral platforms and capability for seamless fusion across modalities of CT, real-time TEE, and fluoroscopy could further optimize procedural guidance and improve procedural efficiency.

### 3D Printing

The benefits of 3D printing for structural heart interventions have been well-described (58). 3D printing may aid in closure device selection and assessment of the interaction between closure device and bioprosthetic or mechanical leaflets, and thus, increase procedural confidence in complex cases. The use of more biologically-textured materials may allow for a more accurate assessment. Limitations include cost and/or need for specialized equipment.

## Conclusion

Advanced cardiac multimodality imaging is a powerful tool that can be successfully applied to transcatheter mitral interventions for improved procedural confidence, procedural success, and outcomes. Multidisciplinary coordination between advanced cardiac imagers and interventionalists is crucial for the evolution of transcatheter mitral procedures.

## Author Contributions

Each author was responsible for outlining and drafting the document, critical review, and figure creation. All authors contributed to the article and approved the submitted version.

## Conflict of Interest

OK has received consulting fees from Boston Scientific and Abbott Structural and Speaker's fees from Edwards Lifesciences. The remaining authors declare that the research was conducted in the absence of any commercial or financial relationships that could be construed as a potential conflict of interest.

## References

[1] Ribeiro AH Wender OC de Almeida AS Soares LE Picon PD. Comparison of clinical outcomes in patients undergoing mitral valve replacement with mechanical or biological substitutes: a 20 years cohort. BMC Cardiovasc Disord. (2014) 14:146. 10.1186/1471-2261-14-146 25326757 PMC4271332

[2] Yoon SH Whisenant BK Bleiziffer S Delgado V Dhoble A Schofer N. Outcomes of transcatheter mitral valve replacement for degenerated bioprostheses, failed annuloplasty rings, and mitral annular calcification. Eur Heart J. (2019) 40:441–51. 10.1093/eurheartj/ehy590 30357365

[3] Nishimura RA O'Gara PT Bavaria JE Brindis RG Carroll JD Kavinsky CJ. 2019 AATS/ACC/ASE/SCAI/STS Expert consensus systems of care document: a proposal to optimize care for patients with valvular heart disease: a joint report of the American Association for Thoracic Surgery, American College of Cardiology, American Society of Echocardiography, Society for Cardiovascular Angiography and Interventions, and Society of Thoracic Surgeons. J Am Coll Cardiol. (2019) 73:2609–35. 10.1002/ccd.28196 31010719

[4] Zoghbi WA Adams D Bonow RO Enriquez-Sarano M Foster E Grayburn PA. Recommendations for noninvasive evaluation of native valvular regurgitation: a report from the american society of echocardiography developed in collaboration with the society for cardiovascular magnetic resonance. J Am Soc Echocardiogr. (2017) 30:303–71. 10.1016/j.echo.2017.01.007 28314623

[5] Zoghbi WA Chambers JB Dumesnil JG Foster E Gottdiener JS Grayburn PA. Recommendations for evaluation of prosthetic valves with echocardiography and doppler ultrasound: a report From the American Society of Echocardiography's Guidelines and Standards Committee and the Task Force on Prosthetic Valves, developed in conjunction with the American College of Cardiology Cardiovascular Imaging Committee, Cardiac Imaging Committee of the American Heart Association, the European Association of Echocardiography, a registered branch of the European Society of Cardiology, the Japanese Society of Echocardiography and the Canadian Society of Echocardiography, endorsed by the American College of Cardiology Foundation, American Heart Association, European Association of Echocardiography, a registered branch of the European Society of Cardiology, the Japanese Society of Echocardiography, and Canadian Society of Echocardiography. J Am Soc Echocardiogr. (2009) 22:975–1014. 10.1016/j.echo.2009.07.013 19733789

[6] Goebel B Heck R Hamadanchi A Otto S Doenst T Jung C. Vena contracta area for severity grading in functional and degenerative mitral regurgitation: a transoesophageal 3D colour Doppler analysis in 500 patients. Eur Heart J Cardiovasc Imaging. (2018) 19:639–46. 10.1093/ehjci/jex056 28444164

[7] Hyodo E Iwata S Tugcu A Arai K Shimada K Muro T. Direct measurement of multiple vena contracta areas for assessing the severity of mitral regurgitation using 3D TEE. JACC Cardiovasc Imaging. (2012) 5:669–76. 10.1016/j.jcmg.2012.03.008 22789934

[8] Zoghbi WA. New recommendations for evaluation of prosthetic valves with echocardiography and doppler ultrasound. Methodist Debakey Cardiovasc J. (2010) 6:20–6. 10.14797/mdcj-6-1-20 20360654

[9] Gunduz S Ozkan M Kalcik M Gursoy OM Astarcioglu MA Karakoyun S. Sixty-four-section cardiac computed tomography in mechanical prosthetic heart valve dysfunction: thrombus or pannus. Circ Cardiovasc Imaging. (2015) 8:e003246. 10.1161/CIRCIMAGING.115.003246 26659372

[10] Mahmood M Kendi AT Ajmal S Farid S O'Horo JC Chareonthaitawee P. Meta-analysis of 18F-FDG PET/CT in the diagnosis of infective endocarditis. J Nucl Cardiol. (2019) 26:922–35. 10.1007/s12350-017-1092-8 29086386

[11] Egbe AC Pislaru SV Pellikka PA Poterucha JT Schaff HV Maleszewski JJ. Bioprosthetic valve thrombosis versus structural failure: clinical and echocardiographic predictors. J Am Coll Cardiol. (2015) 66:2285–94. 10.1016/j.jacc.2015.09.022 26610876

[12] Petrescu I Egbe AC Ionescu F Nkomo VT Greason KL Pislaru C. Long-term outcomes of anticoagulation for bioprosthetic valve thrombosis. J Am Coll Cardiol. (2020) 75:857–66. 10.1016/j.jacc.2019.12.037 32130920

[13] Eleid MF Cabalka AK Malouf JF Sanon S Hagler DJ Rihal CS. Techniques and outcomes for the treatment of paravalvular leak. Circ Cardiovasc Interv. (2015) 8:e001945. 10.1161/CIRCINTERVENTIONS.115.001945 26206850

[14] De Cicco G Russo C Moreo A Beghi C Fucci C Gerometta P. Mitral valve periprosthetic leakage: Anatomical observations in 135 patients from a multicentre study. Eur J Cardiothorac Surg. (2006) 30:887–91. 10.1016/j.ejcts.2006.09.019 17081767

[15] Guerrero M Urena M Himbert D Wang DD Eleid M Kodali S. 1-Year outcomes of transcatheter mitral valve replacement in patients with severe mitral annular calcification. J Am Coll Cardiol. (2018) 71:1841–53. 10.1016/j.jacc.2018.02.054 29699609

[16] Cheung A Webb JG Barbanti M Freeman M Binder RK Thompson C. 5-year experience with transcatheter transapical mitral valve-in-valve implantation for bioprosthetic valve dysfunction. J Am Coll Cardiol. (2013) 61:1759–66. 10.1016/j.jacc.2013.01.058 23500301

[17] Paradis JM Del Trigo M Puri R Rodes-Cabau J. Transcatheter valve-in-valve and valve-in-ring for treating aortic and mitral surgical prosthetic dysfunction. J Am Coll Cardiol. (2015) 66:2019–37. 10.1016/j.jacc.2015.09.015 26516006

[18] Bapat V. Valve-in-valve apps: why and how they were developed and how to use them. EuroIntervention. (2014) 10(Suppl. U):U44–51. 10.4244/EIJV10SUA7 25256331

[19] Pulerwitz TC Khalique OK Leb J Hahn RT Nazif TM Leon MB. Optimizing cardiac CT protocols for comprehensive acquisition prior to percutaneous MV and TV repair/replacement. JACC Cardiovasc Imaging. (2020) 13:836–50. 10.1016/j.jcmg.2019.01.041 31422136

[20] Fedak PW McCarthy PM Bonow RO. Evolving concepts and technologies in mitral valve repair. Circulation. (2008) 117:963–74. 10.1161/CIRCULATIONAHA.107.702035 18285577

[21] Bapat V Pirone F Kapetanakis S Rajani R Niederer S. Factors influencing left ventricular outflow tract obstruction following a mitral valve-in-valve or valve-in-ring procedure, part 1. Catheter Cardiovasc Interv. (2015) 86:747–60. 10.1002/ccd.25928 26386239

[22] Blanke P Naoum C Webb J Dvir D Hahn RT Grayburn P. Multimodality imaging in the context of transcatheter mitral valve replacement: establishing consensus among modalities and disciplines. JACC Cardiovasc Imaging. (2015) 8:1191–208. 10.1016/j.jcmg.2015.08.004 26481845

[23] Mak GJ Blanke P Ong K Naoum C Thompson CR Webb JG. Three-dimensional echocardiography compared with computed tomography to determine mitral annulus size before transcatheter mitral valve implantation. Circ Cardiovasc Imaging. (2016) 9:e004176. 10.1161/CIRCIMAGING.115.004176 27307549

[24] Guerrero M Wang DD Pursnani A Eleid M Khalique O Urena M. A cardiac computed tomography-based score to categorize mitral annular calcification severity and predict valve embolization. JACC Cardiovasc Imaging. (2020) 13:1945–57. 10.1016/j.jcmg.2020.03.013 32417332

[25] Urena M Brochet E Lecomte M Kerneis C Carrasco JL Ghodbane W. Clinical and haemodynamic outcomes of balloon-expandable transcatheter mitral valve implantation: a 7-year experience. Eur Heart J. (2018) 39:2679–89. 10.1093/eurheartj/ehy271 29788044

[26] Blanke P Naoum C Dvir D Bapat V Ong K Muller D. Predicting LVOT obstruction in transcatheter mitral valve implantation: concept of the Neo-LVOT. JACC Cardiovasc Imaging. (2017) 10:482–5. 10.1016/j.jcmg.2016.01.005 26971004

[27] Meduri CU Reardon MJ Lim DS Howard E Dunnington G Lee DP. Novel multiphase assessment for predicting left ventricular outflow tract obstruction before transcatheter mitral valve replacement. JACC Cardiovasc Interv. (2019) 12:2402–12. 10.1016/j.jcin.2019.06.015 31629753

[28] Yoon SH Bleiziffer S Latib A Eschenbach L Ancona M Vincent F. Predictors of left ventricular outflow tract obstruction after transcatheter mitral valve replacement. JACC Cardiovasc Interv. (2019) 12:182–93. 10.1016/j.jcin.2018.12.010 30678797

[29] Praz F Khalique OK Lee R Wu IY Russell H Guerrero M. Imaging in patients with severe mitral annular calcification: insights from a multicentre experience using transatrial balloon-expandable valve replacement. Eur Heart J Cardiovasc Imaging. (2019) 20:1395–406. 10.1093/ehjci/jez050 31220240

[30] Kohli K Wei ZA Yoganathan AP Oshinski JN Leipsic J Blanke P. Transcatheter mitral valve planning and the Neo-LVOT: utilization of virtual simulation models and 3D printing. Curr Treat Options Cardiovasc Med. (2018) 20:99. 10.1007/s11936-018-0694-z 30367270

[31] Babaliaros VC Greenbaum AB Khan JM Rogers T Wang DD Eng MH. Intentional percutaneous laceration of the anterior mitral leaflet to prevent outflow obstruction during transcatheter mitral valve replacement: first-in-human experience. JACC Cardiovasc Interv. (2017) 10:798–809. 10.1016/j.jcin.2017.01.035 28427597 PMC5579329

[32] Khan JM Rogers T Schenke WH Mazal JR Faranesh AZ Greenbaum AB. Intentional laceration of the anterior mitral valve leaflet to prevent left ventricular outflow tract obstruction during transcatheter mitral valve replacement: pre-clinical findings. JACC Cardiovasc Interv. (2016) 9:1835–43. 10.1016/j.jcin.2016.06.020 27609260 PMC5476960

[33] Herrmann HC Szeto WY Litt H Vernick W. Novel use of perfusion balloon inflation to avoid outflow tract obstruction during transcatheter mitral valve-in-valve replacement. Catheter Cardiovasc Interv. (2018) 92:601–6. 10.1002/ccd.27068 28417602

[34] Rahhab Z Ren B de Jaegere PPT Van Mieghem N. Kissing balloon technique to secure the neo-left ventricular outflow tract in transcatheter mitral valve implantation. Eur Heart J. (2018) 39:2220. 10.1093/eurheartj/ehy112 29518218

[35] Guerrero M Wang DD Himbert D Urena M Pursnani A Kaddissi G. Short-term results of alcohol septal ablation as a bail-out strategy to treat severe left ventricular outflow tract obstruction after transcatheter mitral valve replacement in patients with severe mitral annular calcification. Catheter Cardiovasc Interv. (2017) 90:1220–6. 10.1002/ccd.26975 28266162

[36] Guerrero M Dvir D Himbert D Urena M Eleid M Wang DD. Transcatheter mitral valve replacement in native mitral valve disease with severe mitral annular calcification: results from the first multicenter global registry. JACC Cardiovasc Interv. (2016) 9:1361–71. 10.1016/j.jcin.2016.04.022 27388824

[37] Guerrero M Salinger M Pursnani A Pearson P Lampert M Levisay J. Transseptal transcatheter mitral valve-in-valve: a step by step guide from preprocedural planning to postprocedural care. Catheter Cardiovasc Interv. (2018) 92:E185–96. 10.1002/ccd.27128 28557344

[38] Faletra FF Pozzoli A Agricola E Guidotti A Biasco L Leo LA. Echocardiographic-fluoroscopic fusion imaging for transcatheter mitral valve repair guidance. Eur Heart J Cardiovasc Imaging. (2018) 19:715–26. 10.1093/ehjci/jey067 29718146

[39] Krishnaswamy A Tuzcu EM Kapadia SR. Integration of MDCT and fluoroscopy using C-arm computed tomography to guide structural cardiac interventions in the cardiac catheterization laboratory. Catheter Cardiovasc Interv. (2015) 85:139–47. 10.1002/ccd.25392 24403085

[40] Moat NE Duncan A Quarto C. Transcatheter mitral valve implantation: tendyne. EuroIntervention. (2016) 12:Y75–7. 10.4244/EIJV12SYA20 27640042

[41] Meredith I Bapat V Morriss J McLean M Prendergast B. Intrepid transcatheter mitral valve replacement system: technical and product description. EuroIntervention. (2016) 12:Y78–80. 10.4244/EIJV12SYA21 27640043

[42] Kliger C Jelnin V Sharma S Panagopoulos G Einhorn BN Kumar R. CT angiography-fluoroscopy fusion imaging for percutaneous transapical access. JACC Cardiovasc Imaging. (2014) 7:169–77. 10.1016/j.jcmg.2013.10.009 24412189

[43] Eng MH Kherallah RY Guerrero M Greenbaum AB Frisoli T Villablanca P. Complete percutaneous apical access and closure: short and intermediate term outcomes. Catheter Cardiovasc Interv. (2020) 96:481–7. 10.1002/ccd.28731 31957915

[44] Jelnin V Dudiy Y Einhorn BN Kronzon I Cohen HA Ruiz CE. Clinical experience with percutaneous left ventricular transapical access for interventions in structural heart defects a safe access and secure exit. JACC Cardiovasc Interv. (2011) 4:868–74. 10.1016/j.jcin.2011.05.018 21851900

[45] Frerker C Schmidt T Schluter M Bader R Schewel J Schewel D. Transcatheter implantation of aortic valve prostheses into degenerated mitral valve bioprostheses and failed annuloplasty rings: outcomes according to access route and Mitral Valve Academic Research Consortium (MVARC) criteria. EuroIntervention. (2016) 12:1520–6. 10.4244/EIJ-D-16-00209 27998844

[46] Sondergaard L Ussia GP Dumonteil N Quadri A. The CardiAQ transcatheter mitral valve implantation system. EuroIntervention. (2015) 11(Suppl. W):W76–7. 10.4244/EIJV11SWA22 26384199

[47] Shivaraju A Michel J Frangieh AH Ott I Thilo C Schunkert H. Transcatheter aortic and mitral valve-in-valve implantation using the edwards sapien 3 heart valve. J Am Heart Assoc. (2018) 7:e007767. 10.1161/JAHA.117.007767 29982230 PMC6064864

[48] Modine T Vahl TP Khalique OK Coisne A Vincent F Montaigne D. First-in-human implant of the cephea transseptal mitral valve replacement system. Circ Cardiovasc Interv. (2019) 12:e008003. 10.1161/CIRCINTERVENTIONS.119.008003 31510775

[49] Lange R Piazza N. The HighLife transcatheter mitral valve implantation system. EuroIntervention. (2015) 11(Suppl. W):W82–3. 10.4244/EIJV11SWA25 26384202

[50] Ruparelia N Cao J Newton JD Wilson N Daniels MJ Ormerod OJ. Paravalvular leak closure under intracardiac echocardiographic guidance. Catheter Cardiovasc Interv. (2018) 91:958–65. 10.1002/ccd.27318 29024359 PMC6588530

[51] Khan JM Babaliaros VC Greenbaum AB Foerst JR Yazdani S McCabe JM. Anterior leaflet laceration to prevent ventricular outflow tract obstruction during transcatheter mitral valve replacement. J Am Coll Cardiol. (2019) 73:2521–34. 10.1016/j.jacc.2019.02.076 31118146 PMC6664295

[52] Coisne A Pontana F Modine T Sudre A Lancellotti P Hahn RT. Transcatheter mitral valve replacement guided by echocardiographic-CT scan fusion: early human clinical experience. JACC Cardiovasc Interv. (2020) 13:1376–8. 10.1016/j.jcin.2020.02.011 32417097

[53] Schueler R Ozturk C Wedekind JA Werner N Stockigt F Mellert F. Persistence of iatrogenic atrial septal defect after interventional mitral valve repair with the MitraClip system: a note of caution. JACC Cardiovasc Interv. (2015) 8:450–9. 10.1016/j.jcin.2014.10.024 25703879

[54] Malahfji MCS Faza N Chinnadurai P Rehman H Neill J Kleiman N. Clinical utility of CT fusion imaging in guiding transcatheter paravalvular leak closure. J Am Coll Cardiol. (2019) 73(Suppl. 1):1196. 10.1016/S0735-1097(19)31803-0

[55] Basman C Alderwish E Rambhatla T Vaishnav A Kronzon I Mountantonakis SE. A standardized protocol to reliably visualize the left atrial appendage with intracardiac echocardiography: Importance of multiple imaging sites. Echocardiography. (2018) 35:1635–40. 10.1111/echo.14104 30044527

[56] Patti G Mantione L Goffredo C Ussia GP. Intracardiac echocardiography with ultrasound probe placed in the upper left pulmonary vein to guide left atrial appendage closure: first description. Catheter Cardiovasc Interv. (2019) 93:169–73. 10.1002/ccd.27821 30260072

[57] Dhoble A Nakamura M Makar M Castellanos J Jilaihawi H Cheng W. 3D intracardiac echocardiography during TAVR without endotracheal intubation. JACC Cardiovasc Imaging. (2016) 9:1014–5. 10.1016/j.jcmg.2015.08.009 26476502

[58] Tuncay V van Ooijen PMA. 3D printing for heart valve disease: a systematic review. Eur Radiol Exp. (2019) 3:9. 10.1186/s41747-018-0083-0 30771098 PMC6377684

